# YAP1 acts as a negative regulator of pro-tumor TAZ expression in esophageal squamous cell carcinoma

**DOI:** 10.1007/s13402-022-00695-4

**Published:** 2022-08-05

**Authors:** Yi-Zih Kuo, Ya-Rong Kang, Wei-Lun Chang, Lydia Chin-Ling Sim, Tzu-Chin Hsieh, Chu-Han Chang, Yi-Ching Wang, Ching-Jung Tsai, Li-Chun Huang, Sen-Tien Tsai, Li-Wha Wu

**Affiliations:** 1 Department of Otolaryngology, National Cheng Kung University Hospital, College of Medicine, National Cheng Kung University, Tainan, 70101 Taiwan Republic of China; 2 Institutes of Molecular Medicine, College of Medicine, National Cheng Kung University, Tainan, 70101 Taiwan Republic of China; 3 Department of Internal Medicine, National Cheng Kung University Hospital, Tainan, 70428 Taiwan Republic of China; 4 Department of Pharmacology, College of Medicine, National Cheng Kung University, Tainan, 70101 Taiwan Republic of China; 5 Department of Laboratory Science and Technology, College of Health Sciences, Kaohsiung Medical University, Kaohsiung, Taiwan Republic of China

**Keywords:** Esophageal cancer, Squamous cell carcinoma, YAP1, TAZ

## Abstract

**Purpose:**

Although YAP1 and TAZ are believed to be equivalent downstream effectors of the Hippo pathway, differential expression of YAP1 or TAZ suggests distinct functions during cancer progression. The exact role of YAP1 and TAZ in esophageal cancer, the 6th leading cancer-related mortality in the world, remains elusive.

**Methods:**

Following single or double manipulation of YAP1 or TAZ expression, we subjected these manipulated cells to proliferation, migration, invasion, and xenograft tumorigenesis assays. We used RT-qPCR and Western blotting to examine their expression in the manipulated cells with or without inhibition of transcription or translation. We also examined the impact of YAP1 or TAZ deregulation on clinical outcome of esophageal cancer patients from the TCGA database.

**Results:**

We found that YAP1 functions as a tumor suppressor whereas TAZ exerts pro-tumor functions in esophageal cancer cells. We also found a significant increase in TAZ mRNA expression upon YAP1 depletion, but not vice versa, despite the downregulation of CTGF and CYR61, shared targets of YAP1 and TAZ, in xenografted tissue cells. In addition to transcriptional regulation, YAP1-mediated TAZ expression was found to occur via protein synthesis. Restored TAZ expression mitigated YAP1-mediated suppression of cellular behavior. By contrast, TAZ silencing reduced the promoting effect exerted by YAP1 depletion on cellular behaviors. The observed anti-tumor function of YAP1 was further supported by a better overall survival among esophageal cancer patients with a high YAP1 expression.

**Conclusion:**

From our data we conclude that YAP1 functions as a suppressor and negatively regulates pro-tumor TAZ expression via transcriptional and translational control in esophageal cancer.

**Supplementary Information:**

The online version contains supplementary material available at 10.1007/s13402-022-00695-4.

## Introduction

Esophageal cancer is the 8th most common cancer with an estimated 456,000 new cases each year worldwide and is the 6th leading cause of cancer-related mortality [1]. There are two different subtypes, esophageal squamous cell carcinoma (ESCC) and esophageal adenocarcinoma. ESCC has a high prevalence of 60-70% worldwide [2, 3] and often occurs in the upper and middle parts of the esophagus. ESCC is the most common histological form (> 80%) in Eastern Asia [4, 5]. Chronic irritation of the esophagus contributes to the development of esophageal cancer. The major risk factors for esophageal cancer include tobacco use, alcohol abuse, obesity and betel nut chewing depending on the subtype [6, 7]. Surgery, radiation therapy and chemotherapy are the current treatment options for this cancer type. In addition to high recurrence rates, reaching ~40% even after primary tumor resection, the 5-year survival rate for all stages of esophageal cancer remains only 15% [8].

The Hippo pathway, first identified in *Drosophila*, has emerged as a conserved signaling pathway, and has been found to be essential for the proper regulation of organ size and tissue growth [9–11]. Yes-associated protein 1 (YAP1) and its paralog, PDZ-binding motif (TAZ/WWTR1), are two major downstream transcriptional co-activators without DNA binding domains in the Hippo pathway [12–14]. YAP1 and TAZ share 45% amino acid sequence identity with a similar domain organization [15]. They can also shuttle between the cytosol and the nucleus, and regulate the expression of downstream targets by interacting with other transcription factors like TEADs [11, 16]. When the Hippo pathway is on, phosphorylation of YAP1 or TAZ can lead to their cytosolic retention and reduced expression of their respective target genes. Once the pathway is off, unphosphorylated YAP1/TAZ enters the cell nuclei and functions as a transcriptional co-activator to modulate the expression of target genes including CTGF and CYR61 [10, 17, 18]. Dynamic and precise control of YAP1 or TAZ abundancies and their activities is thus crucial to ensure proper physiological regulation and tissue homeostasis.

Aberrant hyper-activation of YAP1 or TAZ causes tissue overgrowth, epithelial-mesenchymal transition, and increases in cell migration and proliferation [19]. Although YAP1 and TAZ are equivalently placed as downstream effectors of the Hippo pathway with oncogenic roles in human cancers, several lines of evidence indicate that YAP1 and TAZ are not functionally reductant and exert distinct functions depending on the cancer types involved [16, 20–23]. First, YAP1 and TAZ knockout mice have been found to exhibit different phenotypes, i.e., YAP1 null mice are lethal at embryonic day 8.5 [24], whereas TAZ null mice are viable [25]. Second, differential levels of YAP1 and TAZ expression in the same cancer type suggest their discrete functions during cancer progression through both shared and unique mechanisms of regulation [26]. Consistent with this notion TAZ, but not YAP1, was found to be predominantly expressed in hepatocellular carcinoma. While attenuating cell growth, TAZ depletion conferred cancer stem cell-like properties, accompanied by increased YAP1 expression, in liver cancer cells [27]. Third, it has been found that YAP1 and TAZ can inter-regulate each other’s protein abundance through TEAD-mediated transcription [16]. Together, it appears that dynamic changes in YAP1 and TAZ expression is essential for the control of cell fate.

Exome sequencing analysis of 113 tumor-normal ESCC pairs revealed mutations of several genes in the Hippo pathway [28], highlighting the importance of the Hippo pathway in esophageal carcinogenesis. Accordingly, two nonsynonymous mutations in FAT4, an upstream activator of the Hippo pathway, were reported to participate in modifying the risk of esophageal cancer [29]. YAP1 and TAZ are the principle effectors of the canonical Hippo pathway [26]. The role of TAZ has been least explored compared to YAP1 in esophageal cancer [30, 31]. Despite the possibility of having an intrinsic mechanism to maintain the total output or activity of the Hippo pathway by inducing a feedback mechanism [16] and their combined activity of being tightly controlled by distinct mechanisms in different cell types [26], most cancer studies nevertheless focused on YAP1 or TAZ alone rather than together. In this report, we focused on the interplay of YAP1 and TAZ and the mechanism underlying in esophageal cancer.

## Materials and methods

### Cell culture

All the cell lines used in this study are ESCC lines. KYSE-70, KYSE-170, TE2 and TE12 were grown in RPMI-1640 medium supplemented with 10% fetal bovine serum, 100 units/ml penicillin and 0.1 mg/ml streptomycin. CE81T-0 and CE81T-4 cell lines with different invasiveness were derived from CE81T/VGH (BCRC, Hsinchu, Taiwan) and propagated as described before [32]. All cells were cultured in a humidified incubator at 37 °C in 5% CO2.

### Lentiviral preparation and transduction

To generate shRNA bearing lentiviruses, control shLuc or shRNA clones targeting at all the variants of YAP1 or TAZ (Table S1) were transfected into 293 T cells using Lipofectamine 2000 (Thermo Fisher Scientific, Waltham, MA, USA). Viral particles in the medium were collected 48 hours after transfection. The titer of viral particles in the medium was measured using the half-maximal inhibitory concentration (IC50) in human lung carcinoma A549 cells. The cells for knockdown were at 40% confluence before infection with shRNA-bearing lentiviruses at the indicated multiplicity of infection (MOI). To generate YAP1 or TAZ overexpressing cells, pLVX vector, pLVX-Flag-YAP1 encoding the isoform YAP1-2α based on the sequence analysis [33, 34] or pLVX-HA-TAZ [14]-bearing lentivirus was generated in the same way as shRNA-bearing lentivirus. The indicated cells at 40% confluence were also infected at the indicated MOI and enriched by puromycin.

### siRNA transfection

Following seeding cells at the indicated concentrations in 3.5 or 6 cm dishes for 24 hrs, they were transfected with TAZ/WWTR1 ONTARGETplus SMARTpool (GE Dharmacon, cat # L-016083-00-0005) or ONTARGETplus siControl (GE Dharmacon, cat # D-001810-10-05) at 100 nM using Lipofetamine 2000. Twenty-four hrs after the transfection, the cells were trypsinized and re-plated for cell-based functional assays on the following two days. The silencing effect was validated by Western blot analysis at 72 hrs post-transfection.

### Trans-well migration or invasion assays

Both assays were performed in triplicate using 24-well Millicell culture inserts with 8 μm-pore polycarbonate membranes. The indicated cells (3 × 10^5^/well) were added to the upper chambers with membranes coated or not with 100 μg/well Matrigel (Corning, NY, USA). The lower chambers were filled with 500 μl growth medium. We used uncoated filters for measuring cell migration and coated ones for measuring cell invasion abilities. After the respective incubation for 16 and 24 hours, the cells that migrated or invaded to the lower surface were stained for 15 minutes with 0.1% crystal violet, and counted in high power fields under a microscope.

### Xenograft tumorigenesis

Male NOD.CB17-*Prkdc*^scid^/NcrCrl (NOD-SCID) mice 6-8 weeks of age were purchased from the National Laboratory Animal Center, housed with a 12-hour light/dark cycle and fed with a sterilized diet and water ad libitum. The animal use for this protocol was reviewed and approved by the Institutional Animal Care and Use Committee at National Cheng Kung University. Vector control, YAP1- and/or TAZ-manipulated cells together with 50 μg Matrigel (Corning, NY, USA) were subcutaneously injected into the flanks of the NOD-SCID mice (5 per group). One week after injection, tumor sizes were measured every 2 days for 33 days. Tumor tissues were harvested at the endpoint for weight measurement and total RNA isolation. All the animal experiments complied with the ARRIVE guides and were carried out in accordance with the National Institutes of Health Guide for care and use of laboratory animals (NIH publication No.8023, revised 1978).

### Inhibitor treatment

For measuring mRNA stability, the cells were exposed for 1 hr. with actinomycin D (ActD, 5 μg/ml) to block RNA synthesis by RNA polymerase II and allowed to recover for 0 ~ 180 min before RNA isolation at the indicated time points for RT-qPCR analysis. As to the protein stability, the indicated cells were treated for 0 ~ 8 hrs with cycloheximide (CHX, 20 μg/ml) before total protein harvest at the indicated time points for Western blot analysis. The half-life of the indicated protein or mRNA was calculated as the time required for reduction of the quantity to half of its initial value. For the remaining inhibitors, the indicated cells were treated for 24 hrs with vehicle (0.1% DMSO) or the indicated inhibitor at the indicated dose before total protein harvest for Western blot analysis.

### Polysome profile analysis

Polysomes were collected using sucrose-gradient centrifugation as described before [35]. The indicated cells were incubated with 10 μg/ml cycloheximide for 10 min in the CO_2_ incubator at 37 °C. Next, cells were washed on ice with phosphate-buffered saline containing 10 μg/ml cycloheximide (CHX), harvested by scraping and spun down at 500 xg for 5 min at 4 °C. The resulting cell pellet was resuspended in 1 ml of lysis buffer (20 mM Tris, pH 7.5, 100 mM potassium chloride, 5 mM magnesium chloride, 0.5% Nonidet P-40, 100 μg/ml CHX, and protease and RNase inhibitors). The resulting lysates were clarified by centrifugation at 12,000 xg for 10 min at 4 °C. To separate polysomes, samples were layered onto a 10–50% sucrose gradient in lysis buffer and centrifuged at 190,000 xg for 90 min. Following polysomal fraction separation, total RNA was extracted from each sucrose gradient fraction using TRIzol (Thermo Fisher Scientific, Waltham, MA, USA) according to the manufacturer’s instructions. A total of 500 ng RNA was reverse-transcribed into cDNA using a High Capacity cDNA Reverse Transcription Kit (Thermo Fisher Scientific, Waltham, MA, USA). Finally, target cDNAs were amplified and their cycle threshold (Ct) determined using a Fast SYBR Green Master Mix (Thermo Fisher Scientific, Waltham, MA, USA).

### Statistical analysis

Two-tailed Student t-tests were used for the comparisons between two groups. One-way analysis of variance (ANOVA) followed by Tukey’s multiple comparison test was used when more than two groups were compared. Data represent mean ± SD (in vitro) or SEM (in vivo) of the experiments. The Kaplan–Meier method and log-rank test were used to compare survival among patient groups. Statistical significance was indicated as *p < 0.05, **p < 0.01 or ***p < 0 .001.

### Online supplementary methods

All other materials and methods are provided in the supplementary information.

## Results

### YAP1 expression is higher than that of TAZ in esophageal cancer cell lines

Although YAP1 and TAZ are paralogs, previous studies revealed distinct roles of either protein in different cell types during animal development [18, 36]. Since both proteins share 46% overall amino acid sequence identity with similar topology [37], we used RT-qPCR and Western blot analysis to, respectively, measure their relative mRNA and protein expression levels in CE81T/VGH, CE81T(1-0), CE81T(1-4), KYSE-70 and KYSE-170 cells. We found that the expression level of YAP mRNA was at least four times higher than that of TAZ in the esophageal cancer lines (Fig. S1a), indicating a dominant role of YAP1 in these cells. CE81T(1-0) and KYSE-170 exhibited relatively high YAP1 protein expression levels compared to the other three cell lines. CE81T/VGH, CE81T(1-0), and KYSE-70 cells exhibited relatively high TAZ protein expression levels among the five tested cell lines (Fig. S1b). Unlike a concordant expression of YAP1 mRNA with its encoded protein with a borderline statistical significance (γ = 0.8489 and *p* = 0.0689), the dis-concordant expression of TAZ mRNA with its protein suggests a complex TAZ regulation in these cells (Fig. S1c). Although both genes share similar upstream regulatory pathways [38], we did not observe a correlation of either gene in mRNA and protein levels in the tested esophageal cancer cell lines (Fig. S1d).

### YAP1 expression deregulation affects esophageal cancer cell proliferation, migration and invasion

**Fig. 1:**
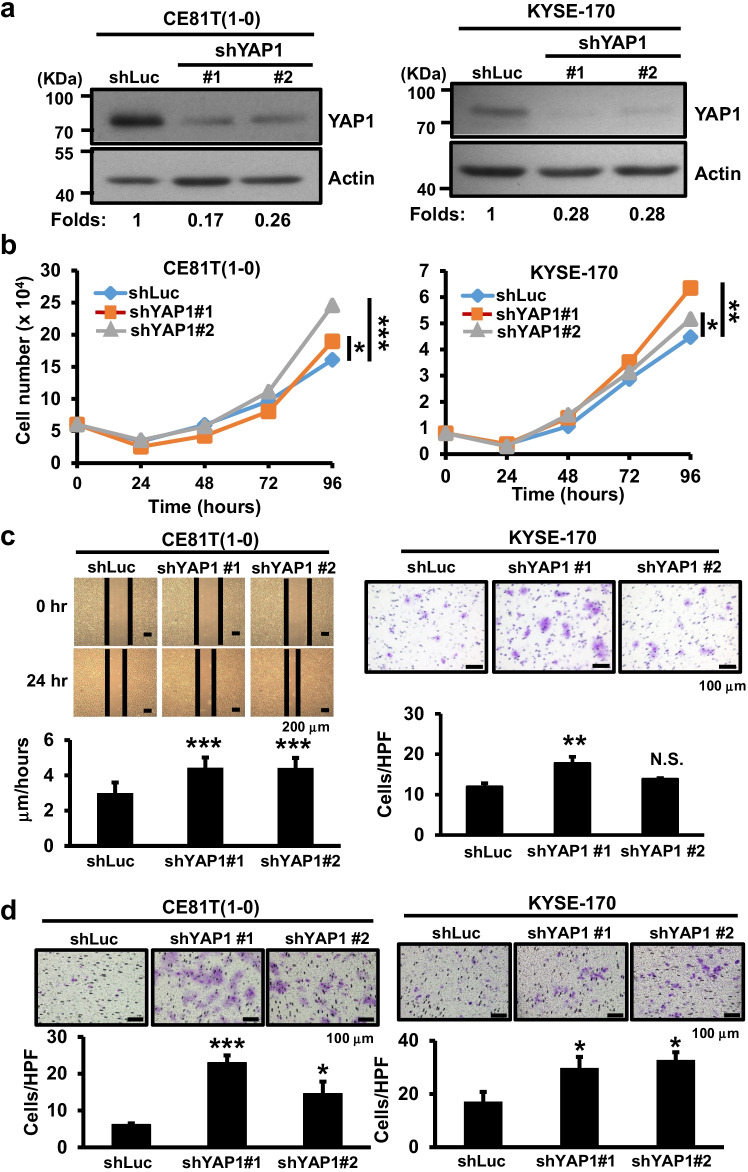

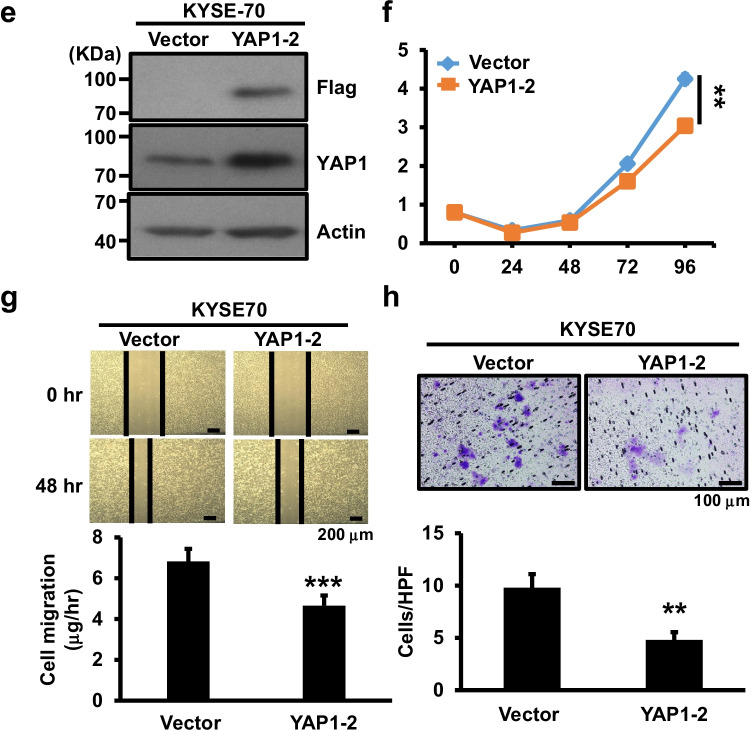
Effect of YAP1 manipulation on esophageal cancer cell proliferation, migration and invasion. **a** YAP1 expression in YAP1-depleted CE81T(1-0) and KYSE-170 cells by Western blotting; β-actin was used as loading control. **b** Live cell numeration of YAP1-depleted CE81T(1-0) and KYSE-170 cells for 4 days after seeding. **c** Migration of YAP1-depleted CE81T(1-0) and KYSE-170 cells measured using scratch wound healing and Trans-well migration assays, respectively. **d** Invasion of YAP1-depleted CE81T(1-0) and KYSE-170 cells determined using a Transwell invasion assay. **e** Western blot analysis of YAP1 expression in the indicated KYSE-70 cells expressing vector or Flag-tagged YAP1-2α. β-actin was used as loading control. Proliferation (**f**), migration (**g**) and invasion (**h**) of vector or stable YAP1-expressing KYSE-70 cells. Each experiment shown is representative of two independent experiments, each performed in triplicates. Data are expressed as mean ± SD. *p < 0.05, **p < 0.01 or ***p < 0.001 versus shLuc. N.S., not significant

YAP1 may function as either a tumor suppressor or promoter [13, 30] and was found to be predominantly expressed in esophageal cancer cells. We first depleted YAP1 expression using shRNA-bearing lentiviral transduction in two relatively high YAP1 expressing cancer cell lines, KYSE-170 and CE81T(1-0) (Fig. S1b). Following confirmation of YAP1 depletion in these lines by Western blotting, we found that YAP1 depletion differentially increased esophageal cancer cell proliferation, migration and invasion abilities in KYSE-170, CE81T(1-0) and TE12 lines compared to to shLuc control cells (Figs. 1a-d and S2), suggesting a tumor-suppressive role of YAP1 in esophageal cancer cells. To confirm the negative role of YAP1 expression in esophageal cancer cells, we performed lentiviral transduction for ectopic expression of Flag-tagged YAP1-2α [13] in relatively low YAP1 expressing KYSE-70 cells followed by various functional assays. Using Western blotting we confirmed the ectopic expression of Flag-tagged YAP1 protein in KYSE-70 cells (Fig. 1e). YAP1 overexpression decreased KYSE-70 cell proliferation, migration and invasion abilities (Fig. 1f-h) compared to vector cells. Together, both the knockdown and overexpression data support a suppressive function of YAP1 in regulating esophageal cancer cell proliferation, migration and invasion.

### TAZ expression deregulation affects esophageal cancer cell proliferation, migration and invasion

**Fig. 2:**
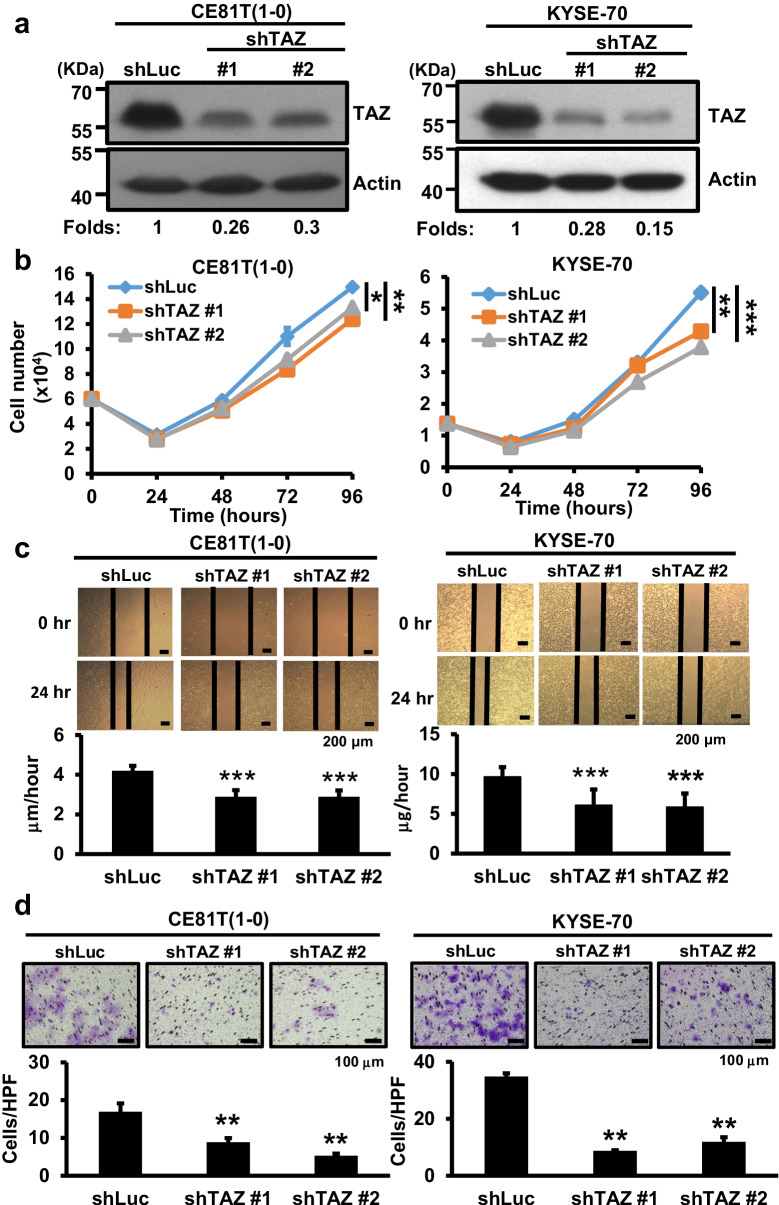

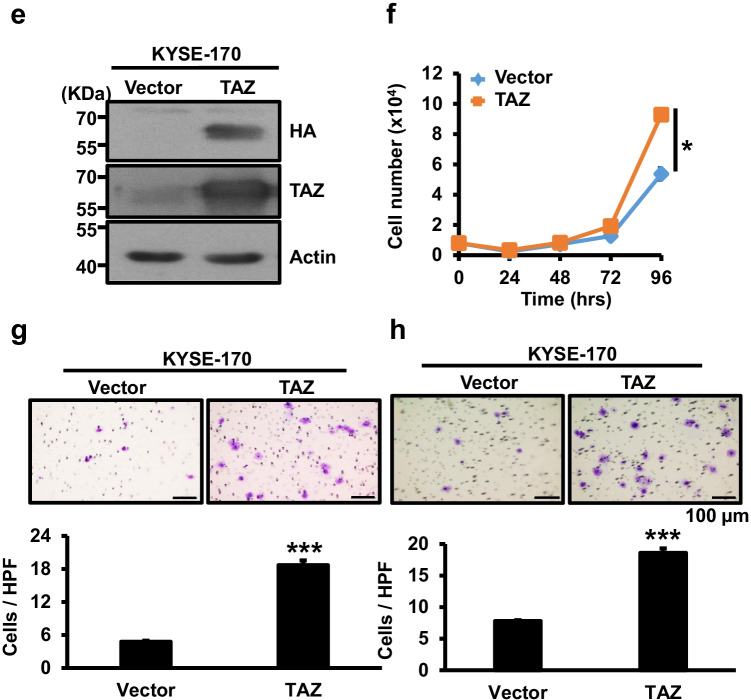
Effect of TAZ expression manipulation on esophageal cancer cell proliferation, migration and invasion. **a** Western blot analysis of TAZ expression in TAZ-depleted CE81T(1-0) and KYSE-70 cells. β-actin was used as loading control. Proliferation (**b**), migration (**c**) and invasion (**d**) of TAZ-depleted CE81T(1-0) and KYSE-70 cells. **e** Western blot analysis of TAZ protein expression in stable KYSE-170 cells expressing vector or HA-tagged TAZ. β-actin was used as loading control. Proliferation (**f**), migration (**g**) and invasion (**h**) abilities of stable KYSE-170 cell lines expressing vector or TAZ. Each experiment shown is representative of three independent experiments, each performed in triplicates. Data are expressed as mean ± SD. *p < 0.05, **p < 0.01 or ***p < 0.001 versus shLuc or vector

TAZ is known to have opposing functions in different cancer types [23, 39]. To examine the role of TAZ in esophageal cancer, we performed TAZ depletion by shRNA-bearing lentiviral transduction in two esophageal cancer cell lines, CE81T(1-0) and KYSE-70, with relatively high TAZ expression levels (Fig. S1b). Using Western blotting we confirmed decreased TAZ protein expression brought about by two shRNA clones (#1 and #2) (Fig. 2a). In contrast to the promoting effect of YAP1 silencing on esophageal cancer cells, TAZ knockdown reproducibly reduced esophageal cancer cell proliferation, migration and invasion in both lines when compared to shLuc control cells (Fig. 2b-d). Next, we ectopically expressed HA-tagged TAZ [14] in low TAZ-expressing KYSE-170 cells by lentiviral transduction for various cell-based assays. Using Western blotting we confirmed the expression of HA-tagged TAZ protein in KYSE-170 cells (Fig. 2e). Ectopic TAZ expression enhanced cell proliferation, migration and invasion (Fig. 2f-h) compared to vector control cells. Both the depletion and overexpression data support a tumor-promoting role of TAZ in esophageal cancer cells.

### Opposing roles of YAP1 and TAZ expression in xenograft tumorigenesis, and the presence of unidirectional induction of TAZ expression in YAP1-depleted esophageal cancer cells

**Fig. 3:**
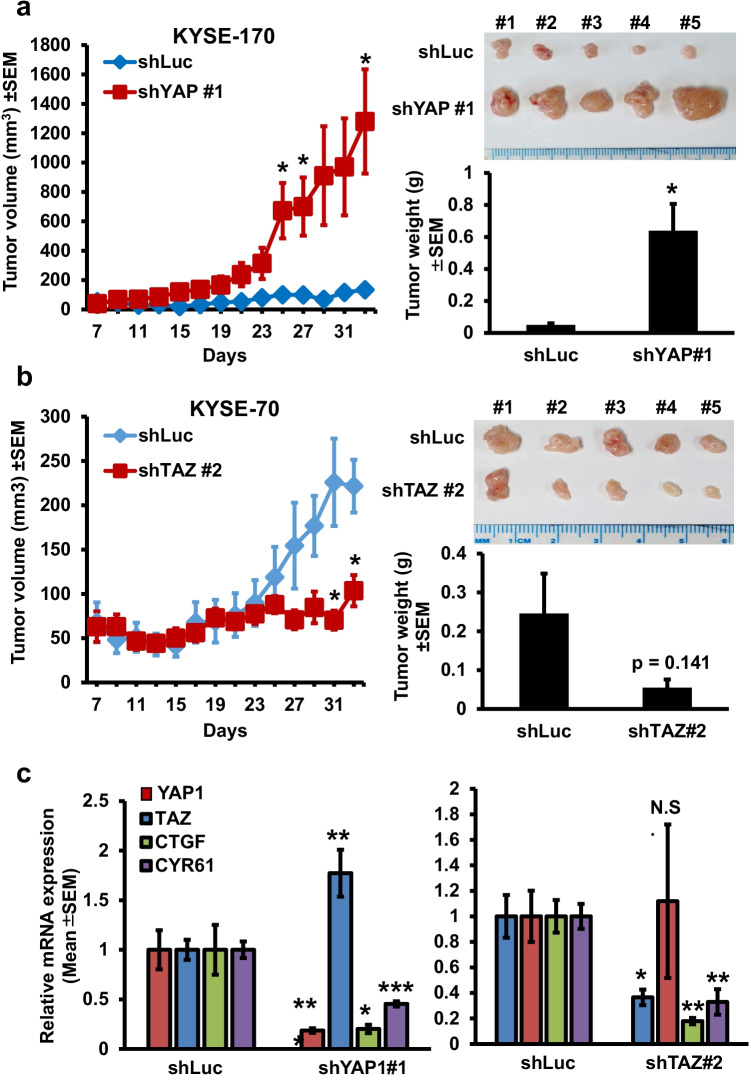
YAP1 and TAZ have opposing functions in xenograft tumorigenesis. **a** Left: Growth curve of tumor xenografts derived from KYSE-170-shLuc and KYSE-170-shYAP1#1 cells implanted in NOD-SCID mice (*N* = 5 per group). Top right: Images of the tumor xenografts derived from KYSE-170-shLuc and KYSE-170-shYAP1#1 cells. Bottom right: The weight of xenografted tumors derived from KYSE-170-shLuc and KYSE-170-shYAP1#1 cells. **b** Left: Growth curve of tumor xenografts derived from KYSE-70-shLuc and KYSE-70-shTAZ#2 cells implanted in NOD-SCID mic (*N* = 5 per group). The images of the tumor xenografts derived from KYSE-70-shLuc and KYSE-70-shTAZ#2 cells (top right) and their tumor weight (bottom right) are shown. **c** Relative mRNA expression of YAP1, TAZ, CTGF and CYR61 in the indicated manipulated cells. Values are all expressed in mean ± SEM (*N* = 5) following normalization with those in the shLuc control. *p < 0.05, **p < 0.01 or ***p < 0.001 versus shLuc

To confirm our in vitro findings, we subcutaneously injected YAP1-depleted KYSE-170 cells (clone #1), TAZ-depleted KYSE-70 cells (clone #2) or shLuc control cells into male NOD-SCID mice. Next, we monitored the tumor volume every two days and harvested the grafted tumors at the endpoints. Consistent with our in vitro findings, we found that YAP1 depletion increased tumor volume and weights (Fig. 3a). By contrast, TAZ depletion reduced tumor volume and weights (Fig. 3b). Although phosphorylation of YAP1 at S127 or that of TAZ at S89 are crucial for their cytosolic retention, respectively [13, 15], we instead detected concordant changes in the level of serine phosphorylation in either protein in the YAP1- or TAZ-manipulated cells by Western blotting (Fig. S3). Since some grafted tumors were small in size (Fig. 3a and b) for both protein and RNA isolation, we examined by RT-qPCR whether expression of their shared targets, CTGF and CYR61, an indicator for the change of nuclear YAP1/TAZ activity [18], was altered by YAP1 or TAZ manipulation. We confirmed mRNA depletion of either gene in the indicated manipulated cells, and correspondingly observed down-regulation of both targets in YAP1- or TAZ-depleted cells (Fig. 3c). Of note, we also detected a significant increase in TAZ mRNA expression in the YAP1-depleted cells but not vice versa (Fig. 3c), suggesting a unidirectional increase of TAZ expression by YAP1 silencing.

### YAP1 negatively regulates TAZ expression partly via translational control in esophageal cancer cells

**Fig. 4:**
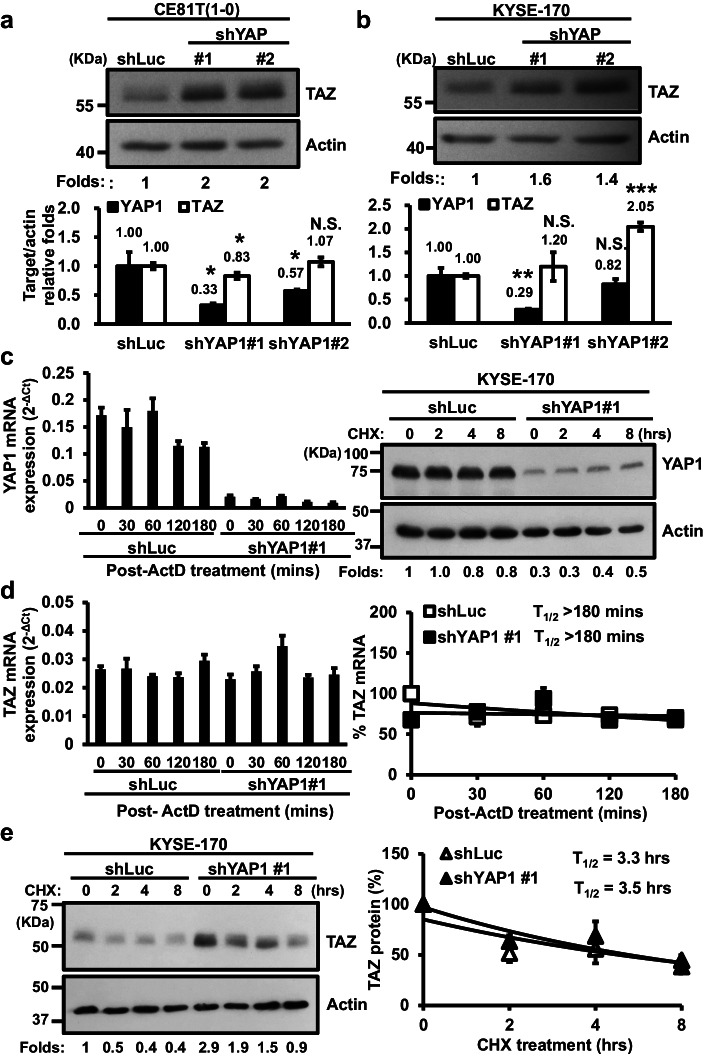

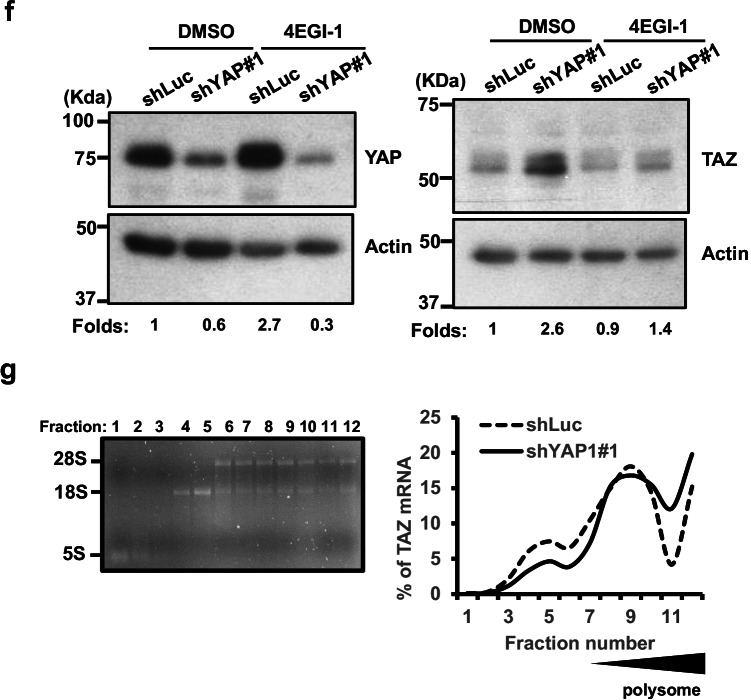
YAP1 depletion enhances TAZ expression in esophageal cancer cells. **a**,** b** Expression of TAZ protein (top) and mRNA (bottom) expression in YAP1-depleted CE81T(1-0) and KYSE-170 cells. Relative folds of TAZ expression are shown at the bottom of the Western blots or the top of the bar graphs. **c** Expression of YAP1 mRNA (left) and protein (right) in YAP1-depleted KYSE-170 cells (shYAP1#1) before and after the indicated treatment. Relative folds of YAP1 expression are shown at the bottom of the blot. **d** Expression (left) and stability (right) of TAZ mRNA in shLuc control and shYAP1#1 cells at the indicated time points following treatment with actinomycin D (ActD). The remaining expression of TAZ mRNA at the indicated time points was quantified as the percent of TAZ mRNA level at 0 min. **e** Expression (left) and stability (right) of TAZ protein in shLuc and shYAP1#1 cells at the indicated time point treated for 0-8 hrs with cycloheximide (CHX). The remaining expression of TAZ protein at the indicated time points was quantified as the percent of TAZ protein level at 0 hr. The graph shows the respective degradation profiles for both conditions (*N* = 3). **f** Expression of YAP1 mRNA (left) and protein (right) in YAP1-depleted KYSE-170 cells (shYAP1#1) before and after the indicated treatment. Relative folds of YAP1 expression are shown at the bottom of the blot. **g** Left: Total RNA from the indicated fractions (1-12) run on an agarose gel and visualized by ethidium bromide staining. Right: Relative distribution of TAZ mRNA on polysome gradients examined by RT-qPCR and expressed as the percentage of total RNA in the indicated fraction

A unidirectional negative control of TAZ protein expression by YAP1 has previously been reported in several mammalian cell types, although the detailed mechanism underlying this effect remains elusive [22]. We first examined the effect of YAP1 silencing on TAZ expression in the manipulated cells by Western blotting and RT-qPCR analyses. We detected a persistent increase in TAZ protein expression without a corresponding increase of its mRNA expression level in most YAP1-depleted esophageal cancer cells, except those depleted by shYAP1#2 (Fig. 4a and b). We next measured the effect of YAP1 overexpression on the expression of TAZ mRNA and protein in YAP1-low KYSE-70 cells by RT-qPCR and Western blotting. We found that ectopic expression of YAP1-2α significantly suppressed TAZ expression at both mRNA and protein levels (Fig. S4). By contrast, TAZ manipulation marginally affected YAP1 protein expression (Fig. S5).

To interrogate whether the YAP1 depletion-mediated increase of TAZ expression was at mRNA or protein expression levels, we treated stable Luc or shYAP1#1 cells with actinomycin D (ActD) or cycloheximide (CHX) at the indicated time points to block the transcription by RNA polymerase II and de novo protein synthesis, respectively. We first confirmed YAP1 mRNA and protein expression depletion in the manipulated KYSE-170 cells (Fig. 4c). The silencing did not affect TAZ mRNA expression in the untreated cells nor its half-life following ActD treatment (Fig. 4d, T_1/2_ > 180 min). The depletion, however, significantly increased the expression of TAZ protein by 2- to 3-fold in YAP1-depleted cells (Fig.4e, left). Following the blockage of de novo protein synthesis by CHX, the half-life of TAZ was marginally affected (Fig.4e, right), indicating the involvement of protein synthesis rather than its stability in YAP1-mediated suppression of TAZ expression.

To examine if translation was involved in the induced expression of TAZ expression upon YAP1 depletion, we first examined the effect of inhibition of the indicated kinases participating in translation on the induced TAZ expression by Western blot analysis. In spite of successful inhibition of these kinases, the pharmacological inhibition of p70S6K, mTOR, ERK1/2 or PI3K failed to attenuate TAZ protein expression in YAP1-depleted cells (Fig. S6). By contrast, the induced TAZ expression was significantly attenuated by a specific inhibitor, 4EGI-1 [40], for cap-dependent translation (Fig.4f). Polysome profile analysis [35], providing information in relation to the number of ribosomes associated with target mRNA, further showed that YAP1 depletion indeed enhanced the enrichment of TAZ mRNA in the polysome (Fig. 4g). Together, YAP1 could regulate TAZ expression via translation control in esophageal cancer cells.

### TAZ expression deregulation attenuates the effect of altered YAP1 expression on cell behavior and target gene expression

**Fig. 5:**
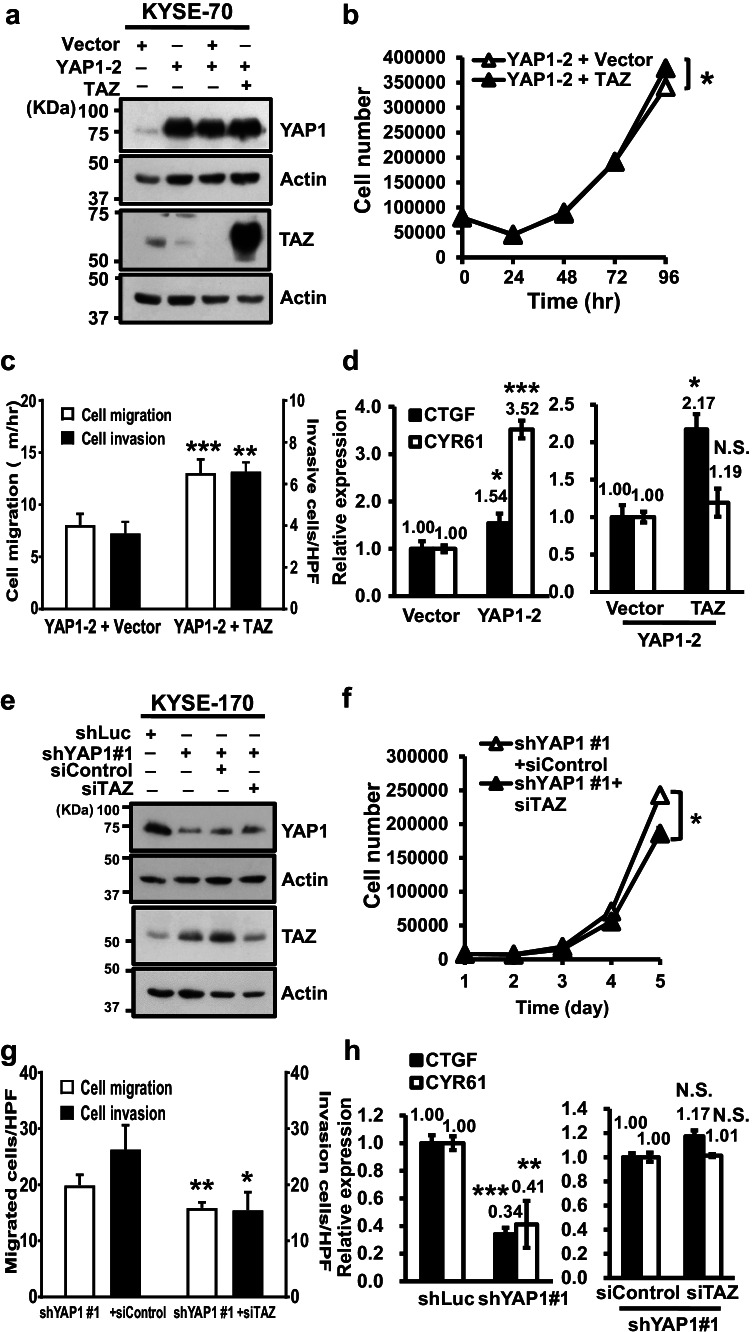
TAZ manipulation attenuated the effect of YAP1 deregulation on cell behavior and target gene expression. Western blot analysis of YAP1 and TAZ expression in the manipulated KYSE-70 (**a**) and KYSE-170 (**e**) cells; β-actin was used as loading control**.** Cell proliferation (**b**), migration and invasion (**c**) of vector and the stable YAP1-expressing KYSE-70 cells. Cell proliferation (**f**), migration and invasion (**g**) of shYAP1-expressing KYSE-70 cells transiently transfected with siControl or siTAZ. The mRNA expression levels of CTGF (black bar) and CYR61 (white bar) were measured in the indicated cells. (**d**, **h**). Each experiment shown is representative of three independent experiments, each performed in triplicates. Data are expressed as mean ± SD. *p < 0.05, **p < 0.01 or ***p < 0.001 versus vector. N.S., not significant

We next set out to assess whether the effect of YAP1 alterations on cellular behavior involves TAZ expression regulation. After having confirmed changes in TAZ expression in YAP1-manipulated cells by Western blotting (Fig. 5a and e), we measured their effect on cellular phenotype and mRNA expression of its target genes, CTGF and CYR61. We found that ectopic TAZ expression increased the proliferation, migration and invasion of YAP1-depleted cells (Fig. 5b-c). Although ectopic YAP1-2 expression increased the mRNA expression levels of CTGF and CYR61, increased TAZ expression enhanced only the expression of CTGF, but not CYR61 (Fig. 5d). Silencing TAZ expression by siRNA, although incomplete, significantly reduced cell proliferation, migration and invasion (Fig. 5f and g) without further suppression of both target genes in YAP1-depleted cells (Fig. 5h). These results support the notion that the promoting effect induced by YAP1 depletion on cell proliferation, migration and invasion is at least in part dependent on the expression of TAZ.

### Clinical relevance of differential expression of YAP1 or TAZ in primary esophageal cancer tissues

**Fig. 6:**
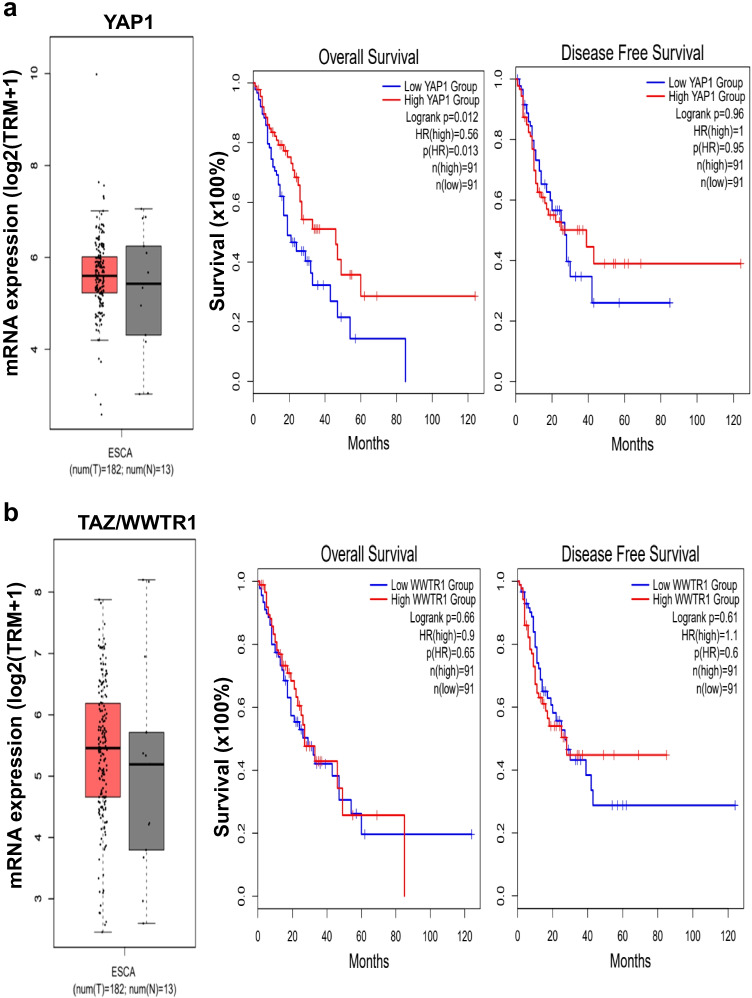
High expression of YAP1 but not TAZ mRNA predicts a better overall survival in esophageal cancer. **a** Left: YAP1 mRNA expression in esophageal tumor (*N* = 182) and normal (*N* = 13) tissues. Red, tumor tissues; Grey, normal tissues. Kaplan–Meier plots indicating the overall survival (middle panel) and disease free survival (right panel) of esophageal cancer patients categorized by high (> median) and low (< median) YAP1 expression (*N* = 91 in each group). **b** Left: TAZ mRNA expression in esophageal tumor (*N* = 182) and normal (*N* = 13) tissues. Red, tumor tissues; Grey, normal tissues. Kaplan–Meier plots indicating the overall survival (middle panel) and disease free survival (right panel) of esophageal cancer patients categorized by high (> median) and low (< median) TAZ expression (*N* = 91 in each group)

To investigate whether the mRNA expression of YAP1 or TAZ was deregulated in primary esophageal cancer specimens, we performed an in silico analysis using publicly available gene expression datasets for esophageal cancer from The Cancer Genome Atlas (TCGA). Further analysis of the TCGA data by Gene Expression Profiling Interactive Analysis 2 (GEPIA2) [41] showed increased expression of YAP1 and TAZ in esophageal cancer tissues relative to normal tissues without reaching statistical significance (Fig. 6, left panels). Next, the patients were then divided into high (> median) and low (< median) groups based on median YAP1 or TAZ mRNA expression in the patient cohort. We found that only the increase of YAP1 expression, but not that of TAZ, statistically predicted a better overall survival (Fig. 6, middle panels), but not a disease-free survival (Fig. 6, right panels) among these patients. These clinical data, together with the cell-based assays and xenograft tumorigenesis, support a negative role of YAP1 expression in esophageal cancer development. The lack of clinical impact of high TAZ expression may be attributed to a much lower level of TAZ expression than that of YAP1 in esophageal cancer cells (Fig. S1a).

## Discussion

We found a higher YAP1 expression than TAZ expression in several esophageal cancer cell lines. Although YAP1 and TAZ are paralogs, we found that YAP1 exhibited a tumor suppressive role, whereas TAZ exhibited a tumor promoting role using both in vitro and in vivo tumorigenesis assays. We also observed an opposite trend in TAZ and YAP1 expression in the YAP1-manipulated cells. The increase in TAZ expression occurred at both mRNA and protein levels rather than at their stability level in the YAP1-depleted cancer cells. Restored TAZ expression attenuated the YAP1-mediated suppression of cell behaviors, whereas TAZ silencing had the opposite effect in YAP1-depleted cells. A strong association of high YAP1 mRNA expression with better overall survival among esophageal cancer patients further supports a negative role of YAP1 in esophageal cancer development. Collectively, we found that YAP1 and TAZ have opposing functions and that YAP1 negatively regulates TAZ expression in esophageal cancer.

In contrast to the reported oncogenic actions of YAP1 overexpression in several cancer types [42], our depletion and ectopic expression studies support a suppressive function of YAP1 expression on esophageal cancer cell proliferation, migration and invasion. Although YAP1 and TAZ depletion reduced the expression of their targets, CTGF and CYR61 [18] in xenografted tumors relative to those in the shLuc control tumors, we also detected a significant increase in TAZ mRNA expression in the shYAP1#1 grafted tumors, but not that of YAP1 in TAZ-depleted tumors. Pharmacological inhibition showed the YAP1 depletion-mediated increase in TAZ expression may occur at the level of protein synthesis. Since we also detected an increase in TAZ mRNA expression in YAP1-depleted grafted tumors and one esophageal cancer line, we cannot completely rule out transcriptional regulation of TAZ expression upon YAP1 depletion.

Our finding is, however, contradictory to the oncogenic function of YAP1 previously reported in esophageal cancer [30, 31]. We noticed that only one cell line, KYSE-170, out of eight esophageal cancer lines was shared among these studies. Moreover, we used shRNA rather than siRNA for the sustained depletion of YAP1 expression. In addition to KYSE-170, the negative effect of YAP1 expression on cell behavior was also detected in three other ESCC lines, CE81T, KYSE-70 and TE12. We further validated that ectopic YAP1 expression attenuated peripheral Ki67+ cell proliferation in xenografted tumors despite unexpected larger cyst-like growth in YAP1-overexpressing xenografts compared to those formed by vector control cells. In support of our findings, a tumor-suppressive role of YAP1 overexpression has been observed in cervical cancer HeLa cells, breast cancer MCF7 cells, glioblastoma D645 cells, and head and neck cancer cells [21, 22].

TAZ was recently identified as a novel bona fide oncogene by promoting cell proliferation, migration, invasion and chemo-resistance in liver cancer cells [23]. Aberrant TAZ overexpression was also found to be associated with tumor size, pathological grade and lymph node metastasis, as well as with an unfavorable prognosis in oral cancer [43, 44]. Although we failed to detect any clinical impact of TAZ expression deregulation on the TCGA-esophageal cancer cohort, likely due to its lower expression than YAP1, TAZ silencing indeed reduced esophageal cancer cell proliferation, migration and invasion. Ectopic TAZ expression had the opposite effect. TAZ depletion also reduced xenograft tumorigenesis and the expression of CTGF and CYR61. In line with this observation, we found that ectopic TAZ expressed restored the suppressive effect of YAP1 on xenograft tumorigenesis. Collectively, our data indicate a pro-tumor role of TAZ in esophageal cancer.

A change in YAP1 abundance/activity can lead to a compensatory change in TAZ expression [22]. Under the condition of YAP1 depletion in esophageal cancer cells and xenograft tumorigenesis, we also detected an increase in TAZ expression. This increase coincided with the pro-tumor effect of ectopic TAZ expression. Moreover, we found that restored TAZ expression attenuated YAP1-mediation suppression of cell proliferation, migration and invasion, as well as CTGF expression. TAZ silencing reduced the promoting effect of YAP depletion on cell behaviors without further attenuating their target gene expression. Pharmacological inhibition studies showed that the YAP1 silencing-mediated increase in TAZ expression may occur at the level of protein expression, although the increase could also result from the increase in TAZ mRNA expression as observed in YAP1-depleted xenografted tumors and one esophageal cancer line. By contrast, no compensatory increase in YAP1 expression was detected in TAZ-depleted cells. The decreased expression levels of CTGF and CYR61, common targets of YAP1 and TAZ [45], were not completely rescued by increased TAZ expression in YAP1-depleted xenografts, supporting distinct functions of YAP1 and TAZ. Caution should thus be taken, especially in the context of pharmaceutical interest in developing inhibitors for YAP1 or TAZ for ant-cancer treatment.

Although a shorter half-life of TAZ compared to that of YAP1 was previously shown to play a role in the YAP1-mediated reduction of TAZ abundance [22], we failed to detect any change in the half-life of TAZ mRNA or protein in the absence of YAP1 expression. Instead, we observed an increased TAZ protein expression in YAP1-depleted cells independent of the activating S6K, ERK1/2, PI3K and mTOR that involve translation regulation. The assembly of the eIF4E/eIF4G complex plays a central role at the level of translation initiation, a rate limiting step in translation. 4EGI-1, an inhibitor for cap-dependent translation [40], abrogated the induced TAZ expression by YAP1. TAZ mRNA was also enriched in the polysomes of YAP1-depleted cells, further supporting the notion that YAP1 negatively regulates TAZ expression partly via translation. Methyltransferase like 3, which recruits initiation factor 3 to the translation initiation complex, was found to enhance the translation of TAZ mRNA containing several N6-methyladenosines (m6A) near the stop codon in the 3′-untranslated region [46]. Whether YAP1 mediates the suppression of TAZ translation also via RNA modification in esophageal cancer cells remains to be established. One limitation is that we could not explain why ectopic YAP1 expression suppressed peripheral Ki67+ cell proliferation, while inducing larger fluid-filled tumor volumes than vector controls in severely immunocompromised NOD-SCID mice.

## Conclusions

Despite similar functions shared by YAP1 and TAZ during the progression of several cancer types [47], our findings indicate that YAP1 and TAZ exhibit opposite functions in mediating esophageal cancer cell behaviors, as well as an unidirectional regulation of TAZ expression by YAP1 in esophageal cancer cells. The ability of YAP1 to regulate TAZ abundance may at least partly account for the contradictory role of YAP1 and TAZ as oncogenes or tumor suppressors in different cellular contexts. In support of our in vitro studies and experimental tumorigenesis, we found that an increase in YAP1 expression predicts a better overall survival among esophageal cancer patients in the TCGA cohort. Although protein expression, especially via cap-dependent translation, is one mechanism involved in the YAP1-mediated suppression of TAZ expression, further studies are needed to assess whether the counteractive YAP1-TAZ axis is a universal or context-dependent phenomenon, and to identify the molecule(s) involved in their regulation.

## Supplementary Information

ESM 1 (PDF 2605 kb)

## Data Availability

All data generated or analyzed during this study are included in this published article/Supplementary Material. Further inquiry can be directed to the corresponding author.
