# Correction to: YAP1 acts as a negative regulator of pro-tumor TAZ expression in esophageal squamous cell carcinoma

**DOI:** 10.1007/s13402-022-00721-5

**Published:** 2022-10-12

**Authors:** Yi-Zih Kuo, Ya-Rong Kang, Wei-Lun Chang, Lydia Chin Ling Sim, Tzu-Chin Hsieh, Chu-Han Chang, Yi-Ching Wang, Ching-Jung Tsai, Li-Chun Huang, Sen-Tien Tsai, Li-Wha Wu

**Affiliations:** 1 Department of Otolaryngology, National Cheng Kung University Hospital, College of Medicine, National Cheng Kung University, Tainan 70101, Taiwan, Republic of China; 2 Institutes of Molecular Medicine, College of Medicine, National Cheng Kung University, Tainan 70101, Taiwan, Republic of China; 3 Department of Internal Medicine, National Cheng Kung University Hospital, Tainan 70428, Taiwan, Republic of China; 4 Department of Pharmacology, College of Medicine, National Cheng Kung University, Tainan 70101, Taiwan, Republic of China; 5 Department of Laboratory Science and Technology, College of Health Sciences, Kaohsiung Medical University, Kaohsiung, Taiwan, Republic of China

**Correction to: Cellular Oncology**

10.1007/s13402-022-00695-4

In this article affiliation 1 was incomplete. The correct affiliation is: Department of Otolaryngology, National Cheng Kung University Hospital, College of Medicine, National Cheng Kung University, Tainan 70101, Taiwan, Republic of China.

The original article has been corrected.

