# Supplementary material for: YAP1 acts as a negative regulator of pro-tumor TAZ expression in esophageal squamous cell carcinoma
Source: Cell Oncol (Dordr). 2022 Aug 5;45(5):893–909. doi: 10.1007/s13402-022-00695-4 (PMC9579103; doi:10.1007/s13402-022-00695-4)

YAP1 acts as a negative regulator of pro-tumor TAZ expression in esophageal squamous cell carinoma

Yi-Zih Kuo[1†], Ya-Rong Kang[2†], Wei-Lun Chang[3], Lydia Chin-Ling Sim[2], Tzu-Chin Hsieh[2], Chu-Han Chang[2], Yi-Ching Wang[4], Ching-Jung Tsai[2], Li-Chun Huang[2], Sen-Tien Tsai[1*], and Li-Wha Wu[2,5*]

[1] Department of Otolaryngology, College of Medicine, National Cheng Kung University, Tainan 70101, Taiwan, R.O.C.

[2] Institutes of Molecular Medicine, College of Medicine, National Cheng Kung University, Tainan 70101, Taiwan, R.O.C.

[3] Department of Internal Medicine, Division of Gastroenterology, National Cheng Kung University Hospital, Tainan 70428, Taiwan, R.O.C.

[4] Department of Pharmacology, College of Medicine, National Cheng Kung University, Tainan 70101, Taiwan, R.O.C.

[5] Department of Laboratory Science and Technology, College of Health Sciences, Kaohsiung Medical University, Kaohsiung, Taiwan, R.O.C.

[†], equal contribution

*, corresponding authors

Please address correspondence to:

Li-Wha Wu, Institute of Molecular Medicine, College of Medicine, National Cheng Kung University, 1 University Rd., Tainan 70101, Taiwan, R.O.C. Tel:+886-6-2353535 ext. 3618; Fax:+886-6-2095845; E-mail: liwhawu@mail.ncku.edu.tw.

Supplementary information

Materials and Methods

RT-qPCR

Total RNA was isolated from half of the frozen tongue tissues or the mouse tumor tissues using TRIzol reagent. One microgram of total RNA was reverse transcribed into cDNA in 20 μl with random hexamers, oligo-dT primers, and MMLV enzyme. Quantitative PCR was conducted by using Fast SYBR Green Master Mix (Thermo Fisher Scientific, Waltham, MA, USA). All reactions were performed in triplicate, and the relative expression of mRNA was calculated using $2^{-\Delta CT}$ method with ACTB as the reference. All primer sequences were listed in Table S1.

Western blotting

Cells were lysed in boiling SDS or RIPA lysis buffer. The protein concentrations were measured by Bradford protein assay (Bio-Rad protein assay, USA). Equal amounts of total proteins were fractionated by SDS-PAGE and blotted onto the polyvinylidene difluoride membrane. The protein blots were hybridized with the indicated primary and then secondary antibodies in 5% non-fat milk, followed by detection with Immobilon Western system (Millipore, Billerica, MA, USA). Primary antibodies include mouse anti-YAP1 antibody (#12395, Cell Signaling Technology), rabbit anti-TAZ antibody (#4883, Cell Signaling Technology), rabbit anti-Phospho-TAZ (Ser89) antibody (#59971), rabbit anti-Phospho-YAP (Ser127) antibody (#4911), and mouse anti-actin antibody (MAB1501, Millipore).

Doubling time

The indicated cells were seeded in triplicate at 10-20% confluence in 24-well plates. We daily enumerated viable cells following trypan blue exclusion for 4 days after seeding.

Scratch wound healing assay

The indicated cells were seeded in duplicate at 90% confluence in 6-well plates pre-coated with 5 μg/ml collagen. Once the cells reached confluence, they were treated with mitomycin C to stop cell proliferation for 24 hrs. We used 200-μl loading tips to generate wounds on the monolayer. Cell migration was monitored and photographed at the indicated hours post-wounding depending on the cell type. The mean distance of ten wound widths along the wound before and after the migration was calculated. The migration rate was the cell migration distance per hour and expressed as Mean ± SD.

Supplementary Table

Tab. S1 shRNA clones in the gene silencing and the primers for qPCR experiments

Supplementary Figures

Fig. S1. The differential expression of YAP1 and TAZ and their relation in esophageal cancer cell lines. The mRNA and protein expression levels of YAP1 or TAZ in five esophageal cancer lines were, respectively, analyzed by (**a**) RT-qPCR and (**b**) Western blotting. β-actin was used as loading control. The immunoblot is representative of three independent experiments. The protein level of YAP1 or TAZ is expressed as mean ± SD of three independent experiments on the bottom of the indicated blot. **c** Pearson correlation showed a borderline correlation of YAP1 mRNA with its encoded protein but no correlation of TAZ mRNA with its encoded protein. **d** Pearson correlation showed no correlation of YAP1 with TAZ both in the mRNA and protein expression levels in in five esophageal cancer lines.

Fig. S2. YAP1 depletion promoted cell proliferation, migration, invasion and TAZ protein expression in TE12 cells

(**a**) Differential expression of YAP1 and TAZ protein in TE2 and TE12 lines relative to those in KYSE-70 and KYSE-170 lines. (**b**) YAP1 and TAZ protein expression in YAP1-depleted TE12 line. Actin, a loading control. (**c**) Proliferation, migration and invasion of YAP1-depleted TE12 cells. Each experiment shown is representative of three independent experiments, each performed in triplicates. Data are expressed as mean ± SD. *$p < 0.05$, **$p < 0.01$, or ***$p < 0.001$ versus vector.

Fig. S3. Concordant changes in the expression of YAP1/TAZ protein expression and the indicated serine phosphorylation in YAP1- or TAZ-manipulated cells. The levels of YAP1 or TAZ proteins and their phosphorylation at the indicated serine residues (S127 for YAP1, and S89 for TAZ) in the YAP1-manipulated (**a**) or TAZ-manipulated (**b**) cells by using Western blotting. Actin was used as loading control. The fold changes for p-YAP1/YAP1 or YAP1/Actin are shown at the bottom of the immunoblots.

Fig. S4. Ectopic expression of YAP1-2α significantly suppressed TAZ mRNA and protein expression. Left: The expression of YAP1 or TAZ mRNA was normalized with ACTB, a loading control, in the indicated KYSE-70 cell clones expressing vector or YAP1-2α. **p<0.01; ***$p < 0.001$ versus vector control. Right: The expression of YAP1/TAZ protein in the indicated KYSE-70 cells. The relative folds of YAP1 or TAZ protein expression are shown on the bottom of the blots.

Fig. S5. Marginal effect of manipulated TAZ expression on YAP1 expression in three esophageal cancer cells. The expression of YAP1 protein in the indicated TAZ-depleted or TAZ-overexpressing cells was detected by Western blot analysis. Actin was used as loading control. Relative folds of YAP1 expression are shown at the bottom of the immunoblots.

Fig. S6. Induced TAZ expression independent of p70S6K, mTOR, ERK1/2 or AKT activating phosphorylation in YAP1 depleted cells. KYSE-170 cells were treated for 24hrs with vehicle (0.1%DMSO), PF470867 (20 μM), rapamycin (1 μM), U0127 (50 μM) or wortmannin (100 nM) to respectively block the activating phosphorylation of p70S6K, mTOR, ERK1/2 and AKT, a substrate of

PI3K, prior their harvest for Western blot analysis. Actin was used as loading control. The induction folds are shown at the bottom of the blots.

Fig. S7. The restored expression of TAZ partially restored YAP1-mediated suppression of cell proliferation in xenograft tumorigenesis.

(**a**) Tumor volume of the indicated xenografts upon subcutaneous injection of the tumor cells into male NOD-SCID mice. (**b**) Two representative image of xenografted tumors (tumors 1 and 2) with fluids in the center. (**c**) Top: representative HE staining showed the peripheral growth but necrotic area in the center of the indicated xenografted tumors. Bottom: the percentage of tumor area in the tumor masses harvested from the mice following HE staining. (**d**) Top: representative image of Ki67+ cells in the tumor xenografts by using IHC staining. Bottom: The percentage of Ki67 positivity in grafted tumor tissues following IHC staining. $p = 0.054$, $p^{**} < 0.01$, $p^{***} < 0.001$, or N.S. versus Vector+vector control. N.S., not significant.

Tab. S1 shRNA clones in the gene silencing and the primers for qPCR experiments

Target Gene	Number	Clone ID	Target Sequence (5’→ 3’)
YAP1 (shRNA)	#1	TRCN0000107265	CCCAGTTAAATGTTCACCAAT
	#2	TRCN0000107269	CGACCAATAGCTCAGATCCTT
TAZ (shRNA)	#1	TRCN0000370007	GCGTTCTTGTGACAGATTATA
	#2	TRCN0000319149	GCGATGAATCAGCCTCTGAAT
YAP1 (qPCR)	F:		TTGGGAGATGGCAAAGACAT
	R:		CTGTGACGTTCATCTGGGAC
TAZ (qPCR)	F:		GGCTGGGAGATGACCTTCA
	R:		AGGCACTGGTGTGGAACTGAC
ACTB (qPCR)	F:		GCAAAGACCTGTACGCCAAC
	R:		TAGAAGCATTTGCGGTGGAC

Figure S1 by Kuo YZ et al

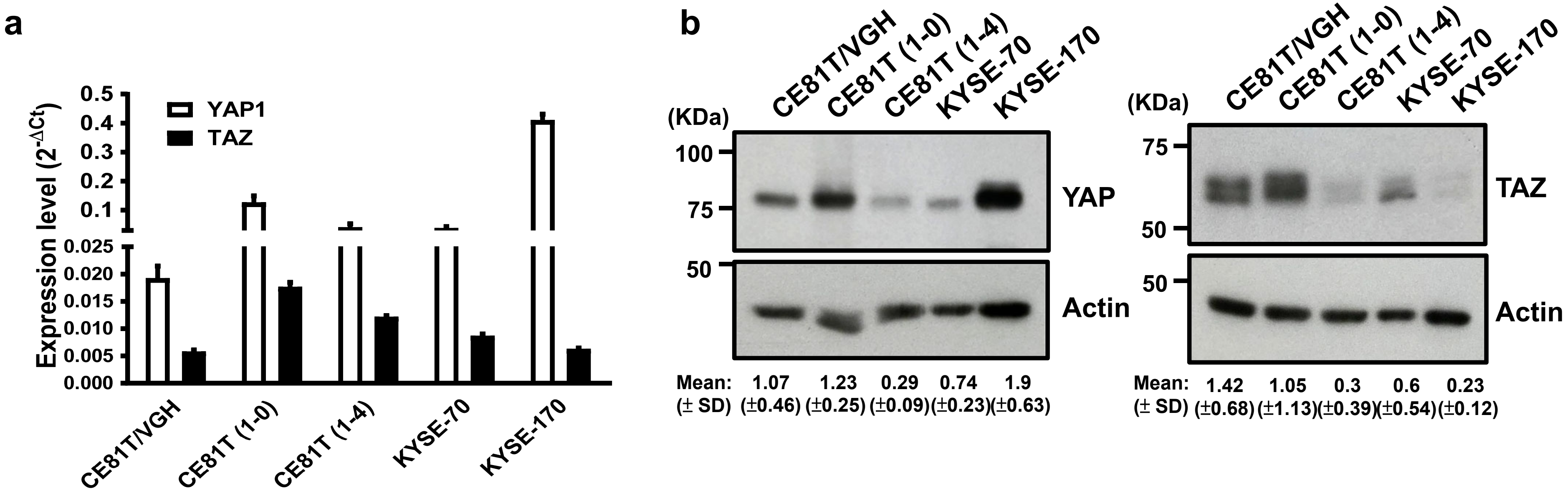

Figure S1 by Kuo YZ et al

c

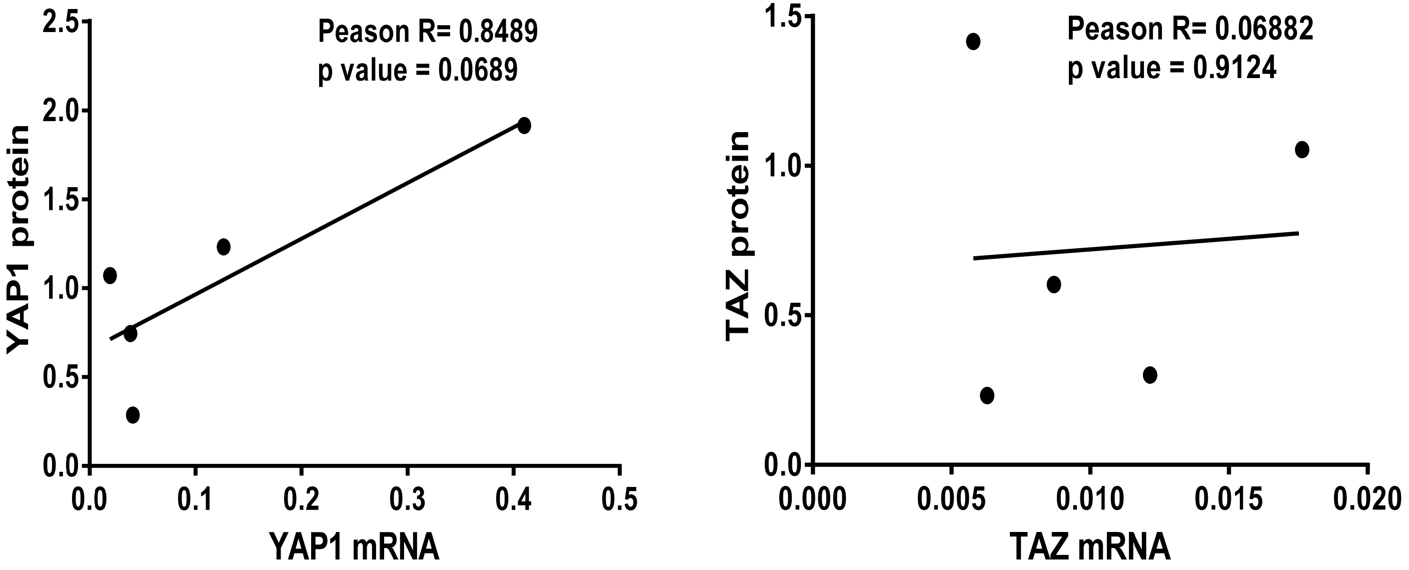

Figure S1 by Kuo YZ et al

d

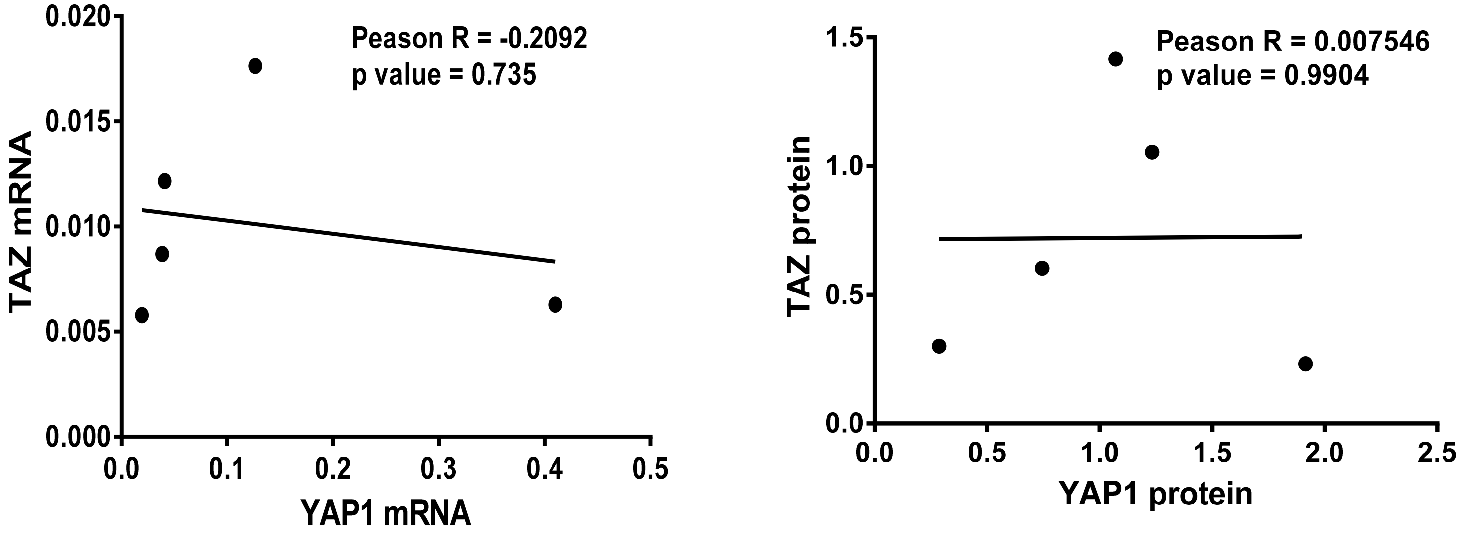

Figure S2 by Kuo YZ et al

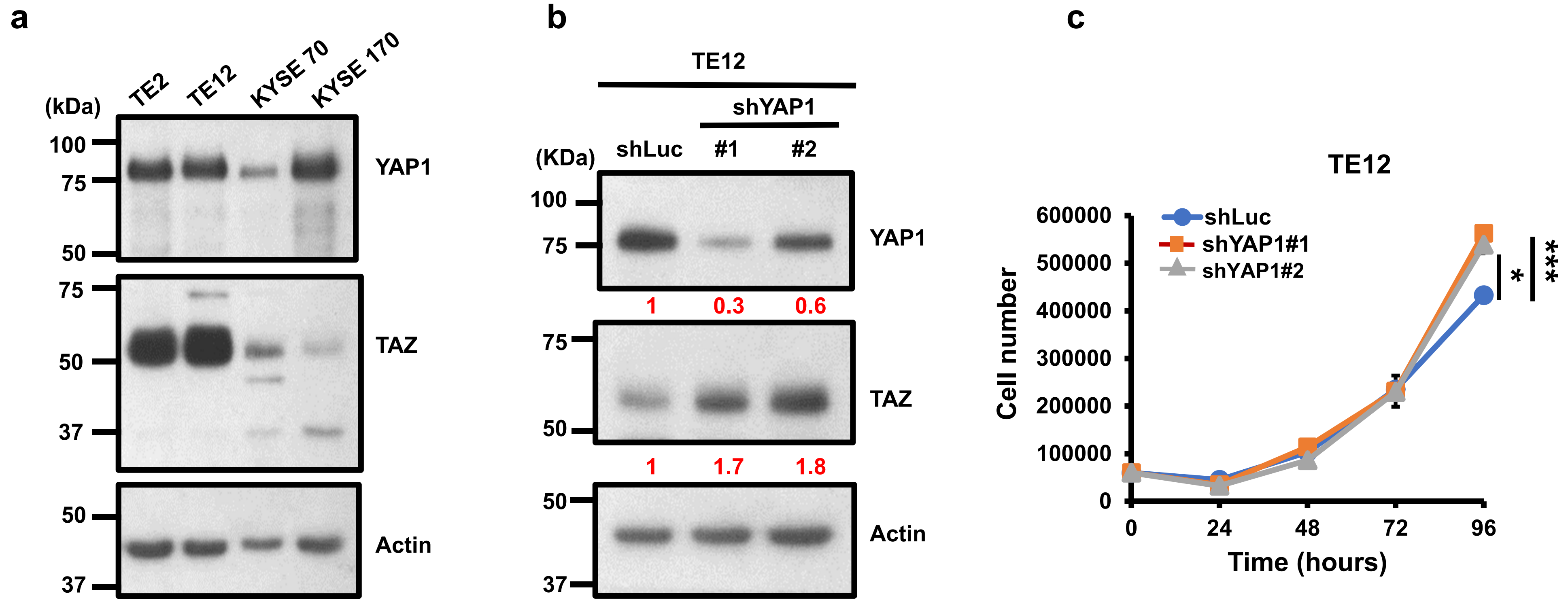

Figure S2 by Kuo YZ et al

d

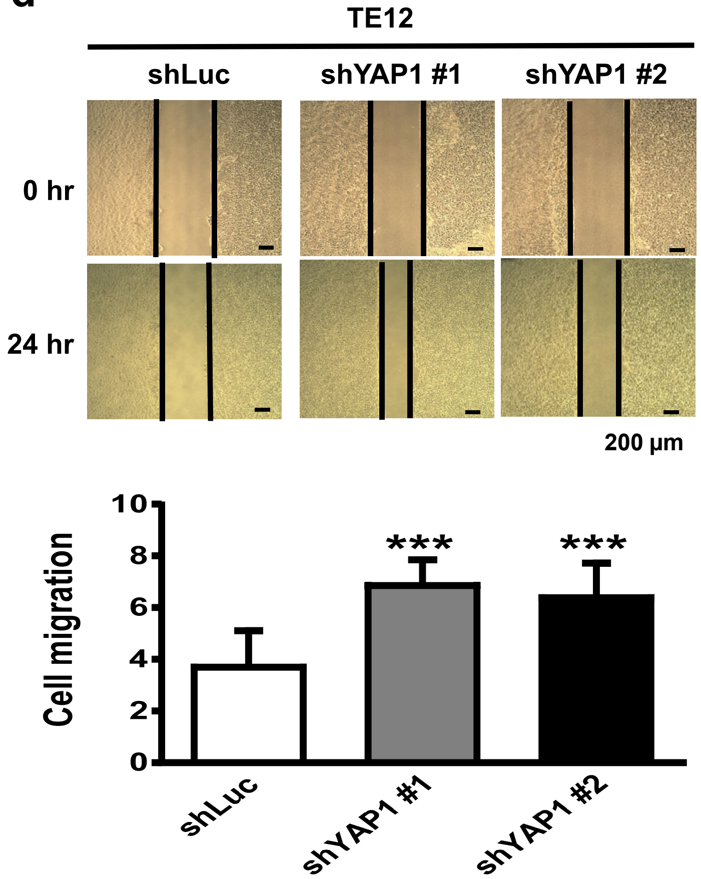

e

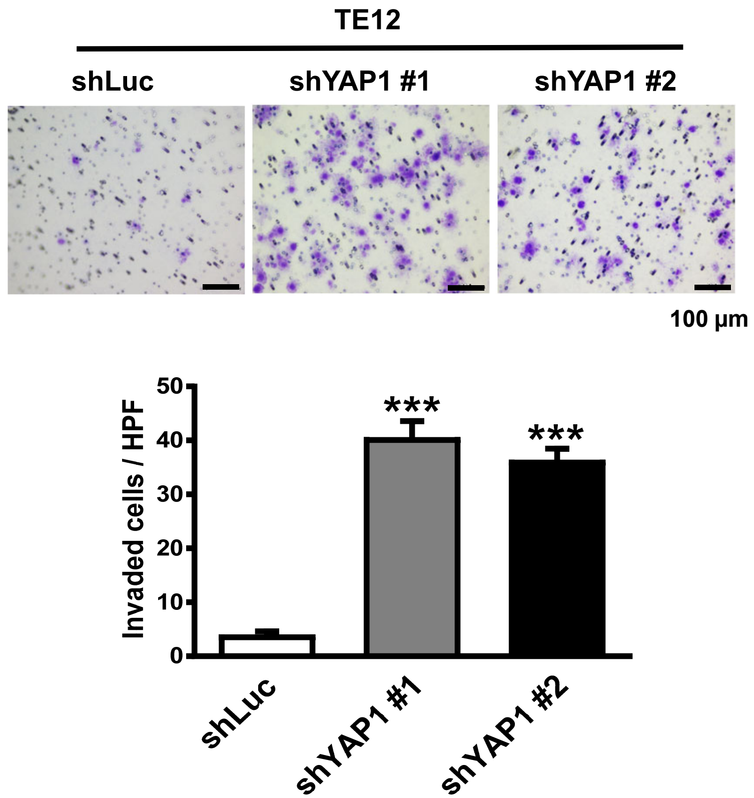

Figure S3 by Kuo YZ et al

a

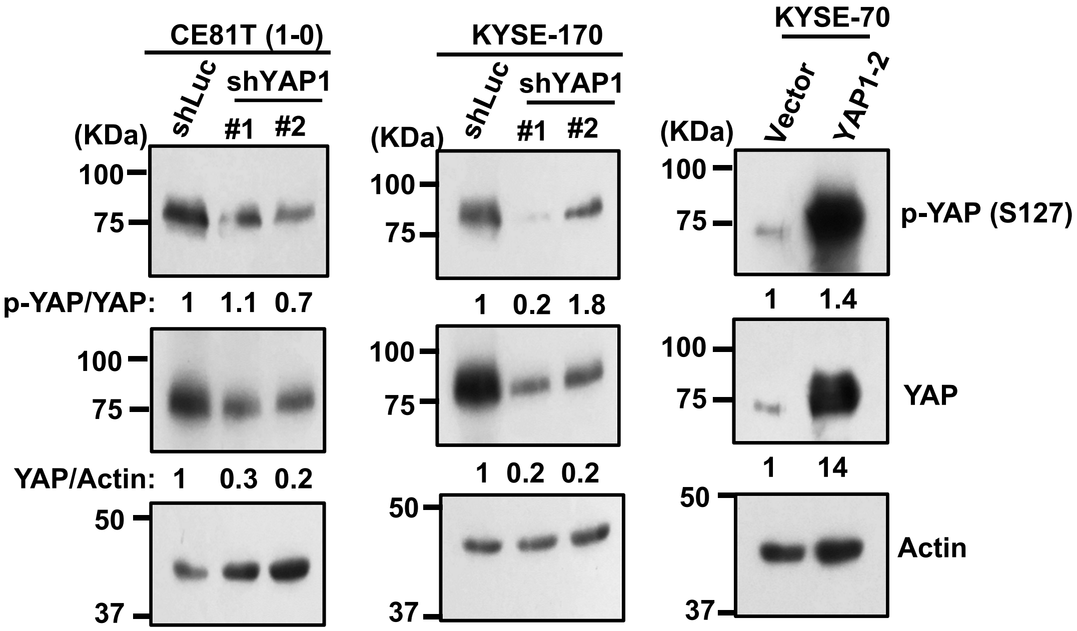

Figure S3 by Kuo YZ et al

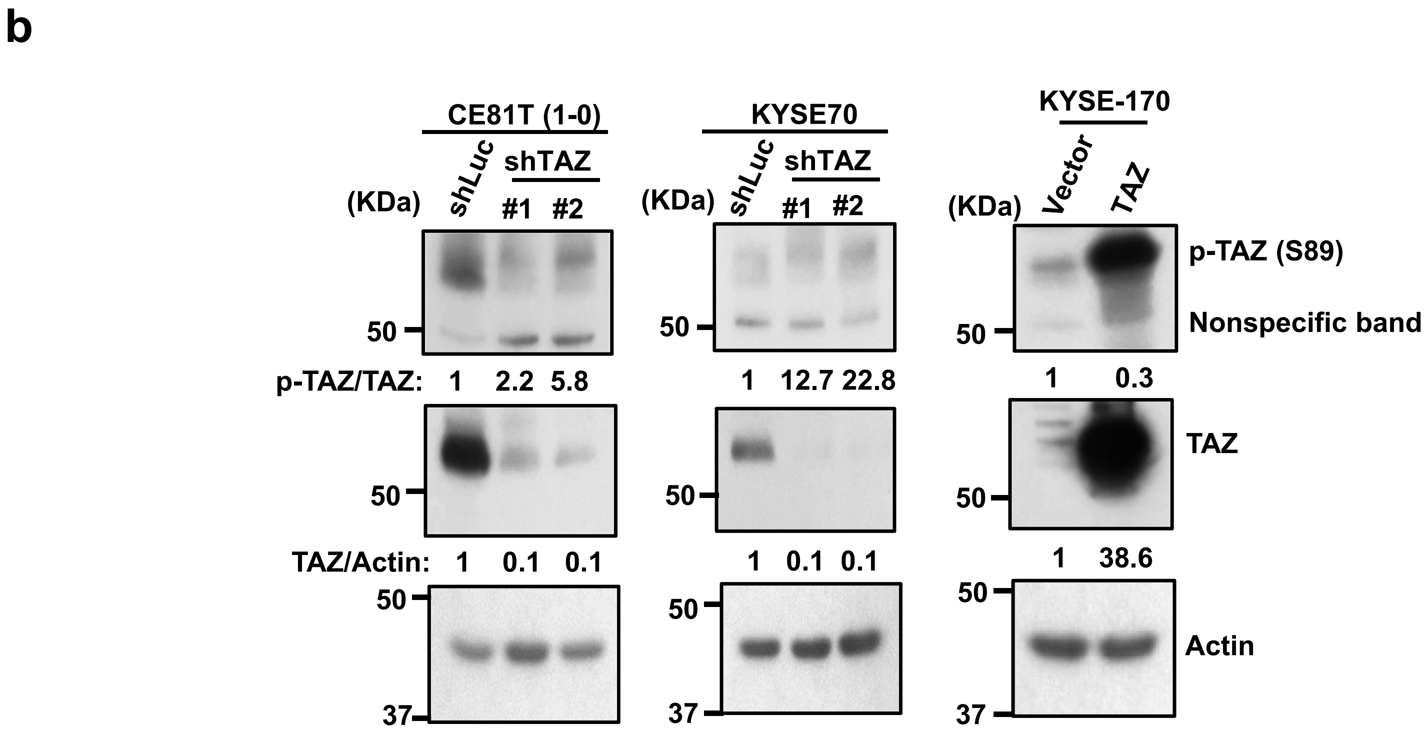

Figure S4 by Kuo YZ et al

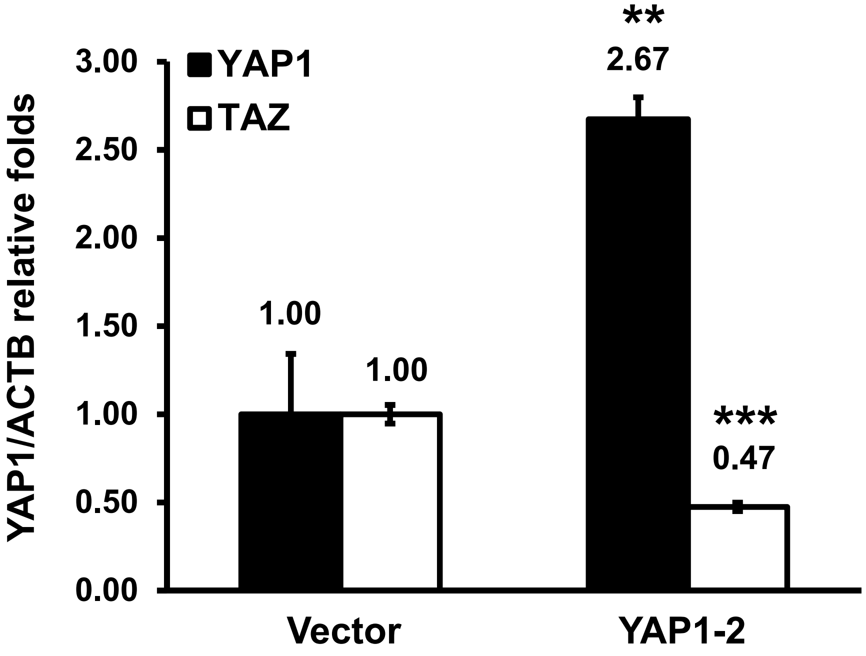

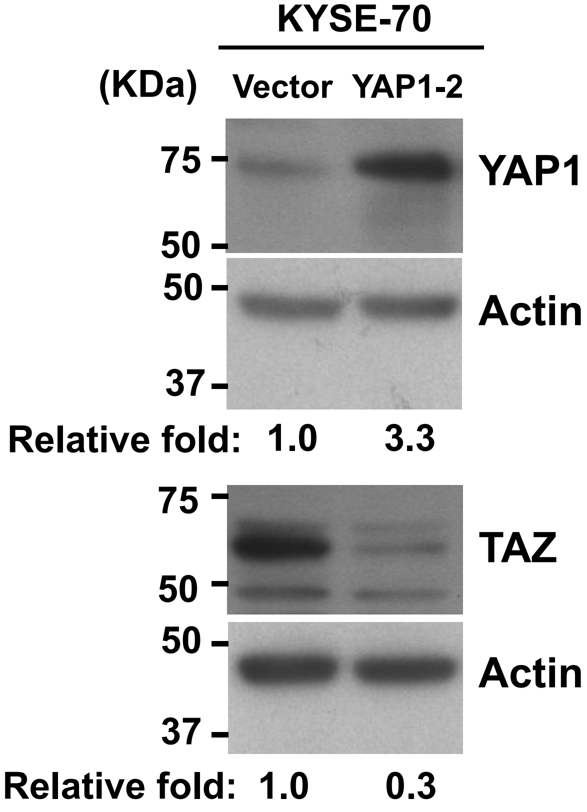

Figure S5 by Kuo YZ et al

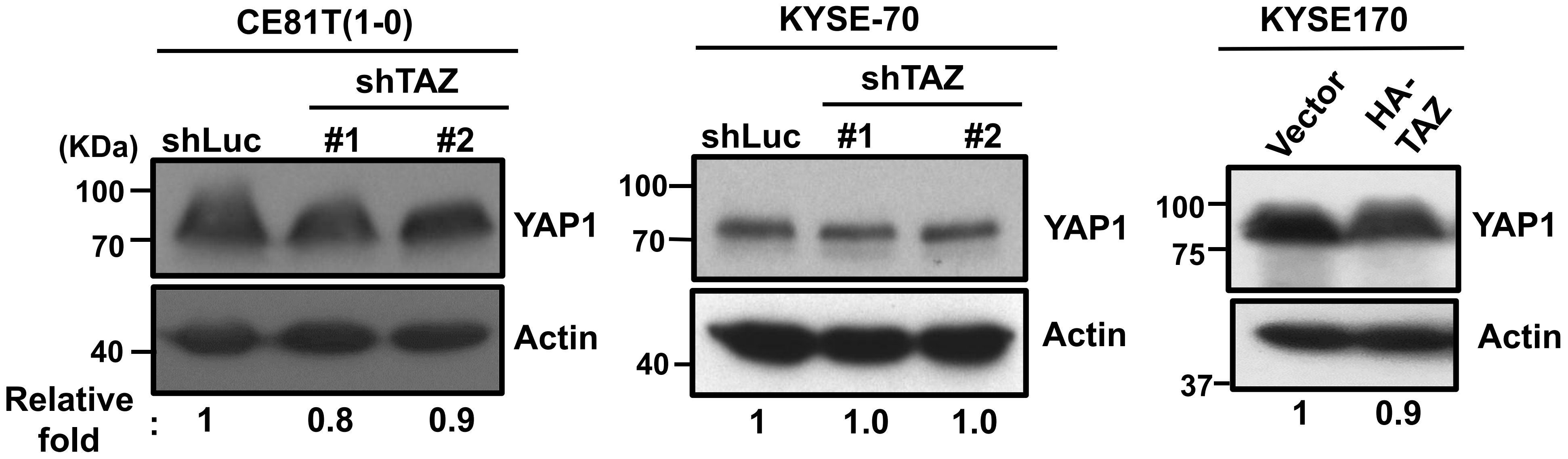

Figure S6 by Kuo YZ et al

a

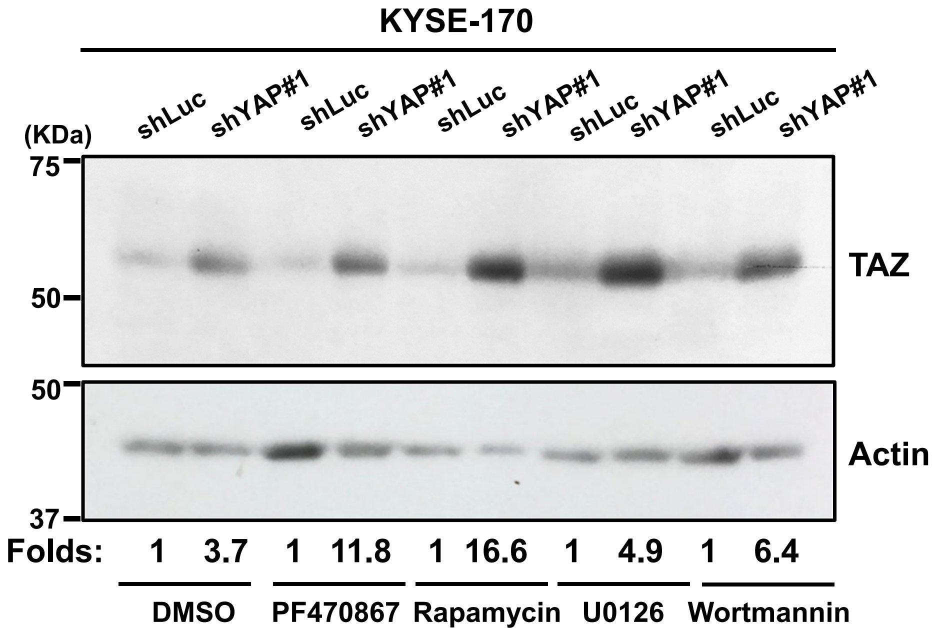

Figure S6 by Kuo YZ et al

b

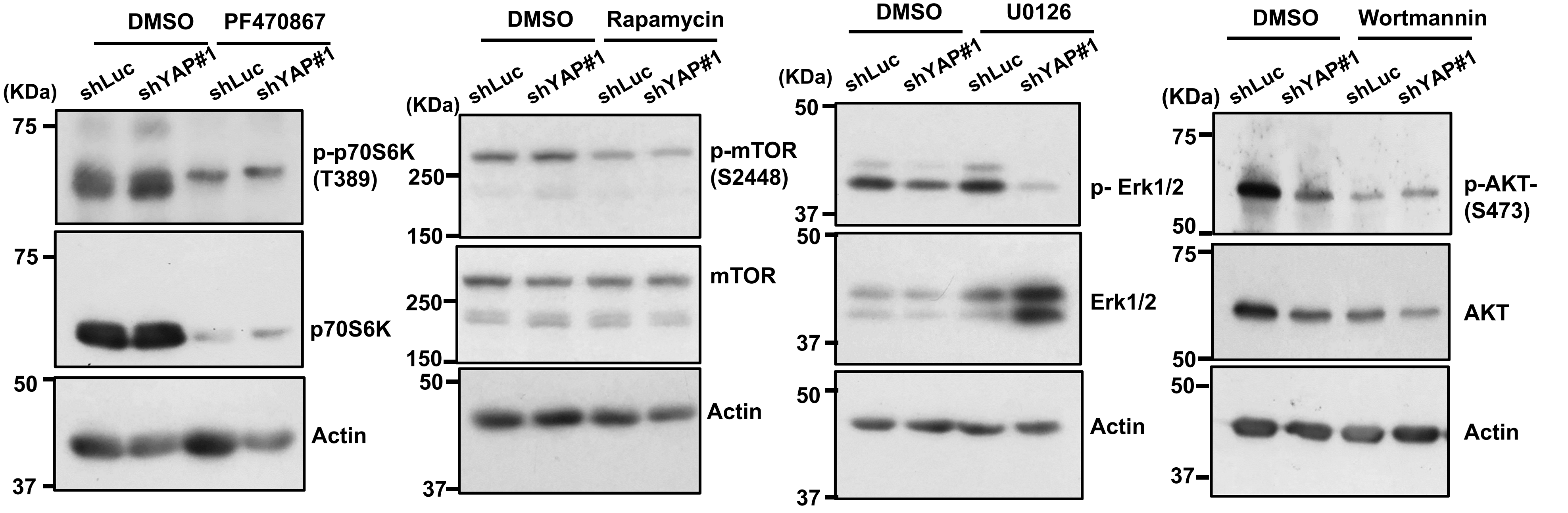

Figure S7 by Kuo YZ et al

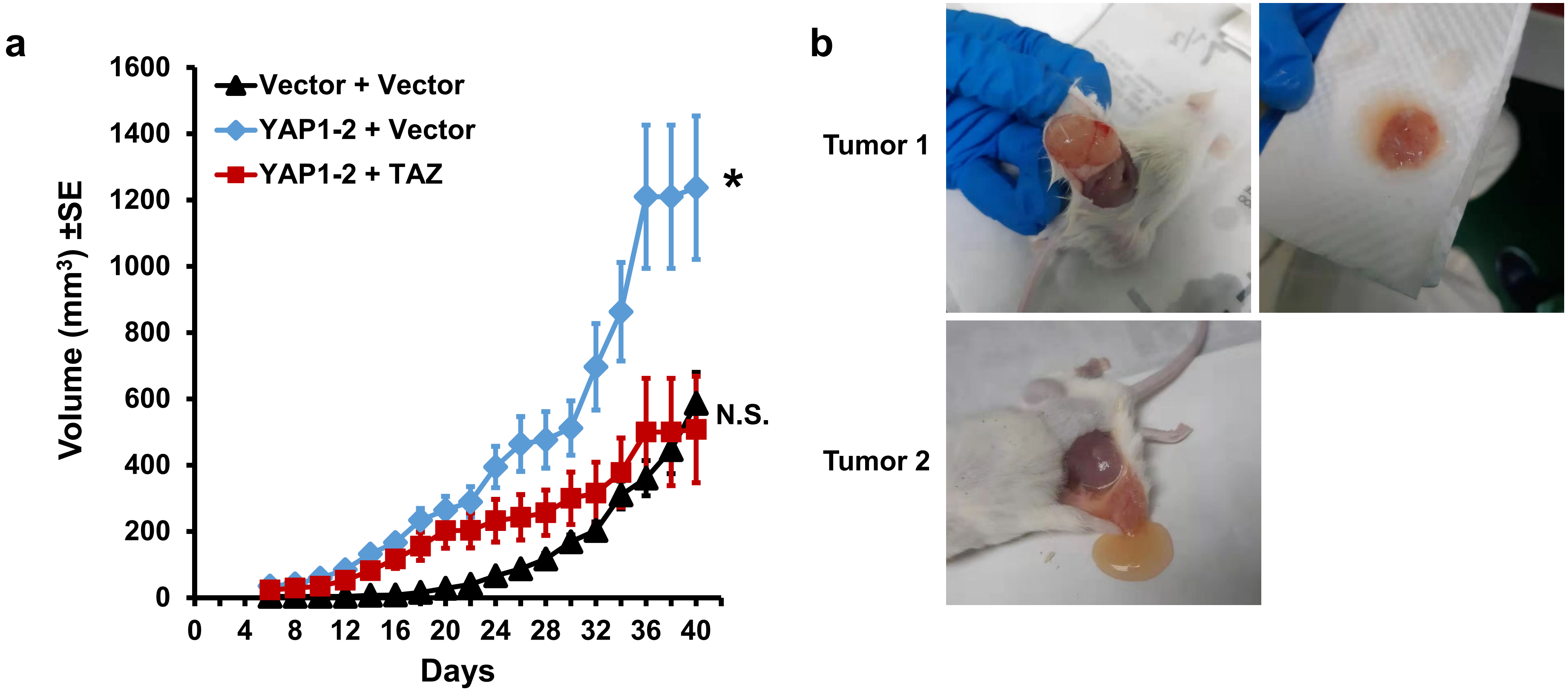

Figure S7 by Kuo YZ et al

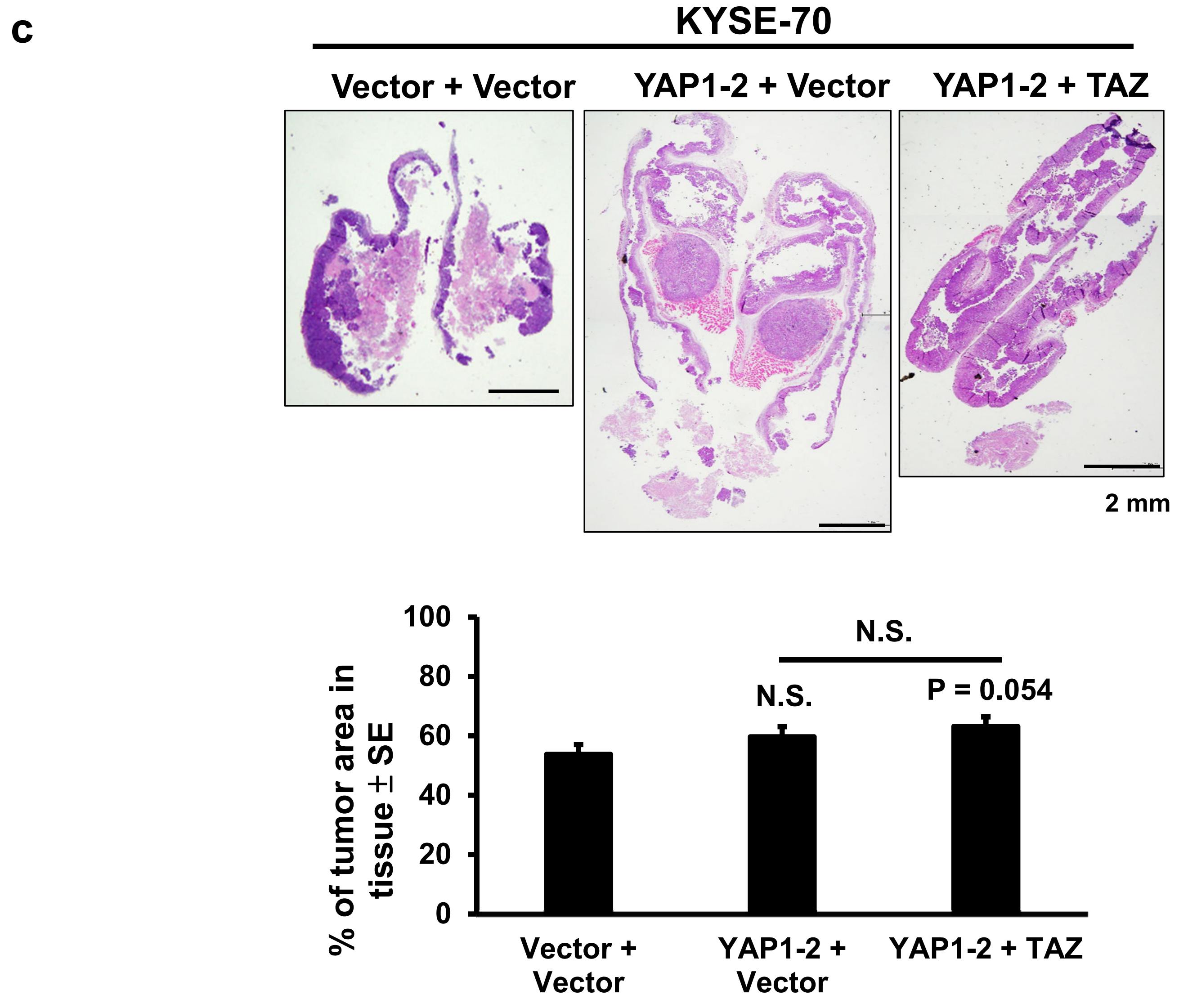

Figure S7 by Kuo YZ et al

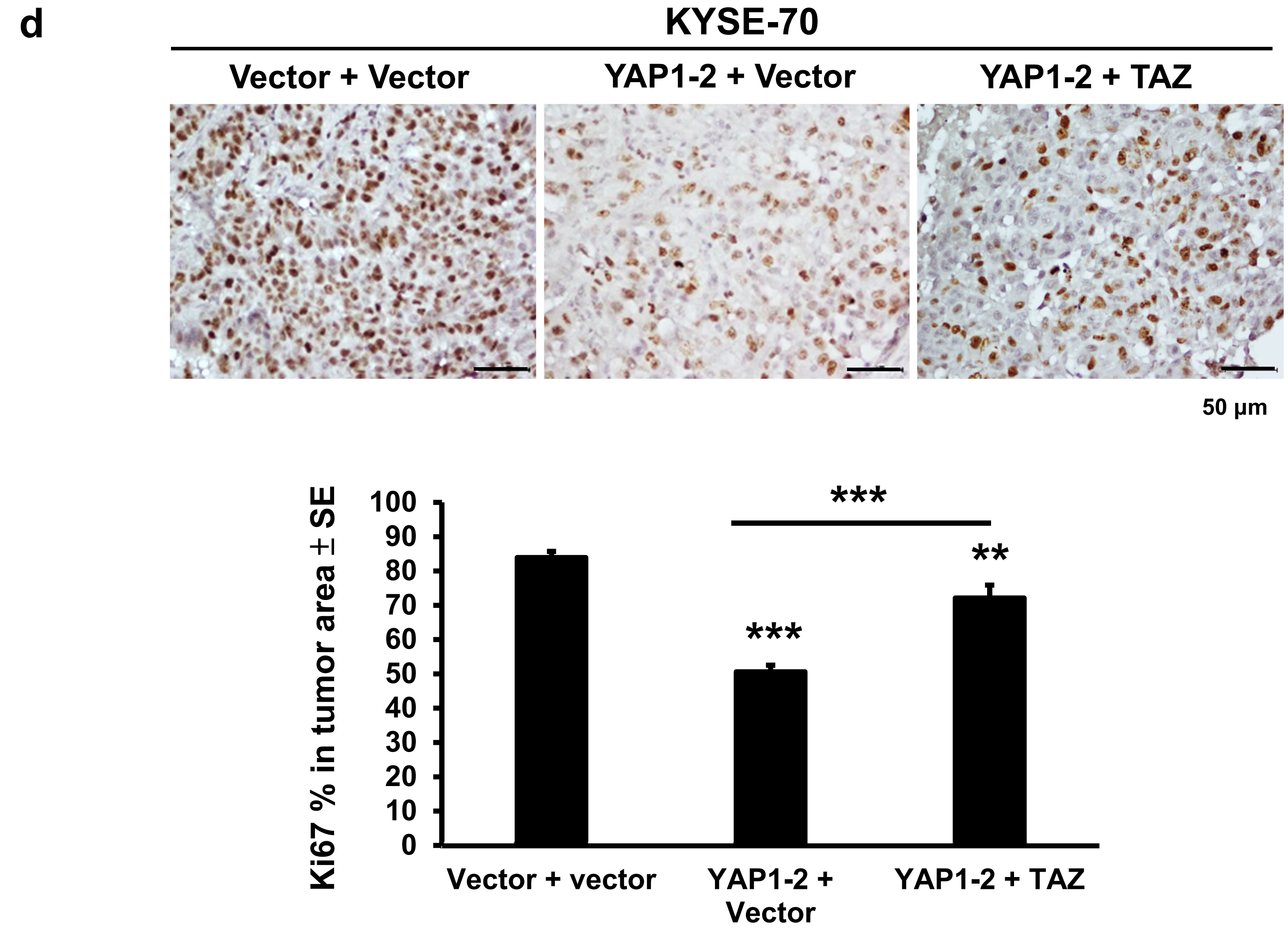

Supplement: Supplementary file 1 — (PDF 2605 kb) [file 13402_2022_695_MOESM1_ESM.pdf]
